# An atypical EF‐hand domain containing resistance protein SRC4 boosts plant immunity in a calcium‐enhanced manner

**DOI:** 10.1111/tpj.71125

**Published:** 2026-09-15

**Authors:** Zikai Zhou, Di Miao, Jie Zhao, Yuxi Zhou, Ting Yan, Niu Niu, Zhuo Bao, Fangjiao Lv, Lu Liu, Ning Xu, Fang Yan, Jun Liu, Hada Wuriyanghan

**Affiliations:** ^1^ Key Laboratory of Forage and Endemic Crop Biology, Ministry of Education, School of Life Sciences Inner Mongolia University Hohhot 010070 China; ^2^ Crop Research Institute, Anhui Academy of Agricultural Sciences Hefei 230031 China; ^3^ State Key Laboratory of Agricultural and Forestry Biosecurity, MOA Key Lab of Pest Monitoring and Green Management, College of Plant Protection China Agricultural University Beijing 100193 China; ^4^ Key Laboratory of State Forestry and Grassland Administration on Sandy Land Biological Resources Conservation and Cultivation Inner Mongolia Academy of Forestry Hohhot 010010 China

**Keywords:** SMV resistance cluster 4, calcium ion, EFh domain, soybean mosaic virus, plant immunity

## Abstract

The typical nucleotide‐binding leucine‐rich repeat (NLR) proteins, such as TIR‐NBS‐LRR (TNL) and CC‐NBS‐LRR (CNL), are known to be engaged in effector‐triggered immunity (ETI). However, several atypical resistance (R) proteins with truncated NLR domains or with nonclassical domains are emerging to be key regulators in plant immunity. SMV resistance cluster 4 (SRC4) is an atypical NLR protein in soybean, distinguished by an N‐terminal Domain (NTD)‐TIR‐shortened NBS oligomerization region (SNOR)‐EF‐hand (EFh) domain architecture. SRC4 lacks both NBS and LRR domains but retains basal antiviral activity through its TIR‐SNOR region, with SNOR contributing to TIR‐dependent antiviral activity and SRC4 self‐association. The EFh domain recognizes coat protein (CP) of soybean mosaic virus (SMV) and serves as a Ca^2+^‐responsive module to distinctly regulate TIR‐SNOR‐mediated immune response in soybean. *SRC4‐*overexpression (Ox‐*SRC4*) soybean plants show SMV resistance without obvious growth penalty. Furthermore, several *SRC4* high‐expression soybean varieties showed stronger SMV resistance and Ca^2+^ responsiveness than the *SRC4* low‐expression soybean varieties. Our study highlights the essential roles of atypical resistance proteins in plant immunity and growth‐defense balance.

## Significance Statement

Calcium signaling contributes to NLR‐mediated immunity, but how calcium levels regulate atypical NLR‐related resistance proteins lacking conventional domains remains poorly understood. Here, we show that soybean SRC4 integrates a Ca^2+^‐responsive EF‐hand module to suppress soybean mosaic virus (SMV) accumulation in a calcium‐enhanced manner, providing evidence that calcium responsiveness can tune antiviral immune output without an obvious growth penalty under the tested conditions.

## INTRODUCTION

Plant immune response conceptually comprises two layers: the pattern‐triggered immunity (PTI) and effector‐triggered immunity (ETI). PTI is the first layer of defense and is activated by the recognition of conserved pathogen‐associated molecular patterns (PAMPs) through pattern recognition receptors (PRRs) (Jones & Dangl, 2006). ETI is mediated predominantly by intracellular nucleotide‐binding leucine‐rich repeat (NLR) receptors, which detect pathogen effectors, often resulting in a hypersensitive response (HR) (Nguyen et al., 2021; Sun et al., 2024). NLRs are categorized into Toll/interleukin‐1 receptor (TIR)‐NLRs (TNLs) and coiled‐coil NLRs (CNLs). Upon activation, CNLs form the so‐called resistosome, a macromolecular complex with calcium‐permeable channel activity, amplifying the immune signaling cascade (Wang et al., 2019). Helper NLRs (hNLRs), such as ADR1 and NRG1, further amplify calcium (Ca^2+^) signals and facilitate systemic acquired resistance (SAR) to ensure whole‐plant immunity (Q. Wang, T, et al., 2024).

TNLs represent a major class of intracellular immune receptors predominantly found in dicotyledonous plants, such as *Arabidopsis thaliana* and *Nicotiana* species (Gantner et al., 2019). The typical TNL protein comprises three conserved structural domains: the TIR domain that mediates downstream signal transduction and immune activation via TIR‐TIR homotypic interactions, the NB‐ARC (NBS) domain that is responsible for ATP/ADP binding and conformational changes, and the leucine‐rich repeat domain (LRR) that confers effector specificity through direct or indirect pathogen recognition (Maruta et al., 2025).

Recent studies have identified some atypical TNLs that exhibit structural and/or functional deviations from typical TNLs. These atypical TNLs often lack the conserved domains or harbor some novel domains other than TIR, NBS, and LRR (Guo et al., 2025; Xi et al., 2022). For instance, certain atypical TNLs lack key conserved domains, such as a partial TIR domain or an altered LRR domain (Liang et al., 2019). Some plants possess TNL variants, which retain only the TIR‐NBS but lack the LRR domain, suggesting that these proteins may not directly recognize pathogen effectors but instead act as downstream immune modulators (Cai et al., 2021; Meyers et al., 2002).

Other than directly triggering ETI‐associated HR, some atypical TNLs are proposed to enhance plant immunity by modulating broader defense networks (Pitsili et al., 2020). Beyond their roles in immunity, specific atypical TNLs have undergone diversification by acquiring functions in regulating plant growth, development, senescence, or adapting to environmental stresses. These observations suggest that atypical TNLs represent an evolutionarily distinct subclass of NLR proteins, with implications for immune signaling plasticity and plant adaptation resilience (Guo et al., 2021; Kulshrestha et al., 2022). Notably, the functional diversification of atypical TNLs is intricately linked to their interactions with conserved signaling hubs. Among these, Ca^2+^‐mediated signaling emerges as a central integrator, bridging pathogen perception to immune execution. Ca^2+^ is a universal and highly versatile secondary messenger, regulating diverse physiological and molecular processes in cells, such as maintaining cellular homeostasis and enabling plants to adapt to diverse environmental conditions (Köster et al., 2022). Under resting conditions, cytosolic Ca^2+^ levels are tightly regulated and are usually at nanomolar levels. However, external stimuli, such as abiotic stress or pathogen invasion, could trigger rapid and transient increases of cytosolic Ca^2+^ levels. This is mediated by plasma and organelle membranes‐located calcium‐permeable channels, which either facilitate Ca^2+^ influx or release Ca^2+^ from intracellular stores, respectively (El Kasmi, 2021). These Ca^2+^ signals are decoded by sensor proteins, such as calmodulins (CaMs), calcium‐dependent protein kinases (CDPKs), and calcineurin B‐like proteins (CBLs), which activate downstream signaling pathways that regulate cellular responses essential for plant growth and survival (Locci & Parker, 2024). The dual roles of Ca^2+^, both as an immune trigger and as a homeostasis regulator, are exemplified in NLR‐mediated immunity. Upon pathogen perception, intracellular Ca^2+^ levels are increased rapidly, leading to the activation of CDPKs, which in turn modulate the downstream NLR immune response (Negi et al., 2023; Yuan et al., 2022). For example, studies on *Arabidopsis thaliana* RPM1 and RPS2 have shown that Ca^2+^ influx enhances NLR oligomerization and signal transduction (C. Wang, T, et al., 2024). In tobacco (*Nicotiana benthamiana*), Ca^2+^ influx is essential for the activation of the NLR receptor NRG1, which forms resistosomes to mediate cell death and antiviral defense (Jacob et al., 2021). These findings reveal a conserved role of Ca^2+^ in modulating NLR immune activation.

Upon pathogen recognition, rapid Ca^2+^ influx often serves as an early signal linking pathogen perception to downstream defense responses (Negi et al., 2023). Of the immune mechanisms regulated by Ca^2+^ signaling, the NLR‐mediated pathway is particularly important (Huang et al., 2023). However, sustained Ca^2+^ influx poses a risk to cellular signal homeostasis, as prolonged NLR activation can lead to growth inhibition, leaf chlorosis, and reduced crop yield (Adachi et al., 2023; Liu et al., 2020). To mitigate the cost of excessive immune activation, plants have evolved a mechanism to precisely regulate the activation of NLRs, where Ca^2+^ plays an essential role. For example, NRG1 and ADR1 helper receptors form Ca^2+^ channels and their activation is regulated by negative feedback from Ca^2+^ signaling (Jacob et al., 2021). Similarly, ZAR1 pentamers function as Ca^2+^ channels and they are ubiquitinated and degraded after short activation, effectively shutting down Ca^2+^ signaling to prevent prolonged ion influx (Bi et al., 2021). Additionally, reactive oxygen species (ROS) interact with Ca^2+^ signaling components, which may regulate the closure of Ca^2+^ channels to restore cellular Ca^2+^ homeostasis during later stages of NLR immunity (Stael et al., 2015).

The balance of growth and immunity represents a fundamental challenge in plant biology. Immune responses, particularly those mediated by NLRs, often act at a significant metabolic cost, which diverts resources from normal plant growth. Overactivation of immune responses would negatively affect plant development and reduce overall fitness (López‐Márquez et al., 2023). Recent studies have highlighted the pivotal role of Ca^2+^ in mediating the balance of growth and immune activation. For example, vacuolar Ca^2+^‐proton exchangers (CAXs) are critical for restoring cellular Ca^2+^ homeostasis by rapidly removing excessive cytosolic Ca^2+^ following immune activation (Lenzoni et al., 2018). This process ensures transient cellular Ca^2+^ spikes to trigger defense and does not impose prolonged growth penalties. Such regulatory mechanism indicates Ca^2+^ signaling acting as a central integrator of growth and defense in plants.

In this study, we report the *SMV Resistance Cluster* (*SRC*) 4 on chromosome 16, which encodes atypical NLRs, the immune response of soybean. SRC4 contains a unique EF‐hand Ca^2+^‐binding domain to replace the typical LRR domain (Yan et al., 2022). SRC4 can initiate robust defense without incurring growth penalties. Importantly, overexpression of *SRC4* in soybean (Ox‐*SRC4*) enhances resistance to SMV but does not cause the fitness costs that are typically associated with NLR‐mediated immunity. Here, the EFh domain plays a critical role in modulating SRC4 activity, allowing it to regulate the trade‐off between growth and defense, revealing an interplay between Ca^2+^ signaling and atypical NLR proteins in plant immunity.

## RESULTS

### SRC4 is an atypical Ca^2+^‐dependent R protein with antiviral activity

**Figure 1:**
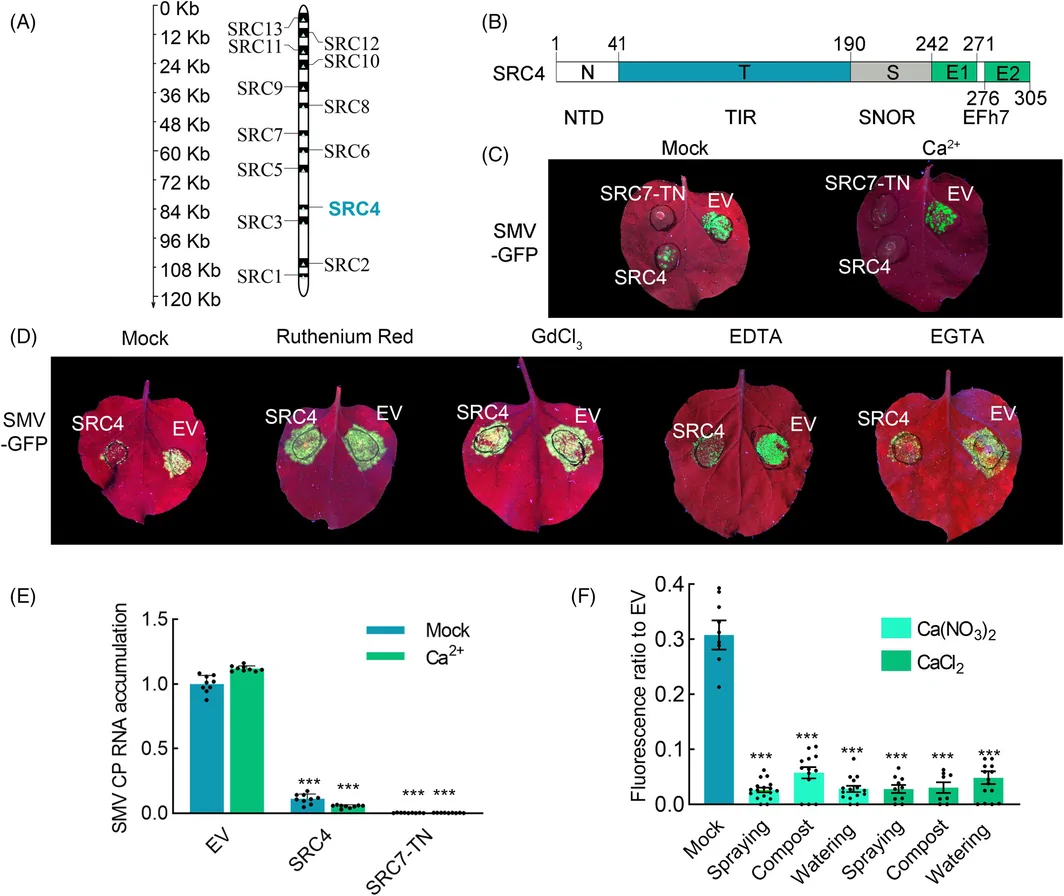
Ca^2+^ enhances SRC4‐mediated antiviral activity. (A) Genomic location of *SRC4* in the soybean (*Glycine max*) SMV resistance cluster (SRC). SRC4 is located on chromosome 16 from 37 160 311 to 37 269 281 bp. (B) Domain structure of SRC4. N: NTD (1–41 aa); T: TIR (42–190 aa); S: SNOR, (191–242 aa); E1 and E2: EFh includes E1 (243–271 aa) and E2 (272–305 aa) subdomains. (C) Antiviral activity of SRC4 is Ca^2+^ dependent. *N. benthamiana* leaves were infiltrated with *Agrobacterium tumefaciens* GV3101 (OD_600_ = 1.0) carrying *SRC4* expression construct and then were infected with SMV‐GFP. Ca^2+^ (10 mM CaCl_2_) was sprayed on leaves. GFP was visualized under handheld UV lamp (wavelength: 365 nm) at 4 dpi (days post infiltration). SRC7‐TN served as a positive control for SMV resistance, EV: empty vector was used as a negative control. Ca^2+^: CaCl_2_ treatment, Mock: ddH_2_O treatment, *n* ≥ 15. (D) Effect of Ca^2+^ inhibitors and chelators on SRC4 antiviral activity. The experiments were performed as same as in (C). Ruthenium Red, GdCl_3_, EDTA, and EGTA were all formulated into 10 mM aqueous solutions and were sprayed on leaves. (E) RT‐qPCR analysis of SMV CP RNA accumulation in EV, SRC4, and SRC7‐TN‐expressing leaves under Mock or Ca^2+^ treatment. Data are presented as means ± SD. Statistical differences were analyzed by one‐way ANOVA, *** indicates *P <* 0.001 (*n* = 9). (F) Effect of different Ca^2+^ supplementation methods on SRC4‐mediated disease resistance. Mock: ddH_2_O treatment. Ca(NO_3_)_2_ and CaCl_2_ were used as Ca^2+^ source in the three supplementation methods summarized in this panel. *N. benthamiana* leaves were infiltrated with *Agrobacterium tumefaciens* GV3101 (OD_600_ = 1.0) carrying pNC‐Cam2304‐35S‐SRC4 and then infected with SMV‐GFP. GFP fluorescence was captured under a handheld UV lamp (wavelength: 365 nm) at 4 dpi and analyzed with Fiji software. The chart shows the fluorescence intensity ratio of experimental groups to EV group. Data are presented as Mean ± SEM. Statistical differences were analyzed by one‐way ANOVA, *** indicates *P <* 0.001 (*n* ≥ 15).

In the previous study, we identified a SMV resistance cluster (SRC) in soybean, among which SRC7 exhibits a broad‐spectrum antiviral activity. SRC7 is an atypical TNL with the structure of TIR‐NBS‐BSP, in which BSP is a novel basic secretory protein domain (Yan et al., 2022). The *SRC* gene cluster is located in the 109‐kb region (37160311–37269281 base pair, bp) of soybean chromosome 16. The region contains 13 genes encoding proteins with TIR structural domains (*SRC1*–*SRC13*). Of these proteins, SRC1, SRC4, SRC6, SRC7, and SRC8 possess antiviral activities (Figure 1A) (Yan et al., 2022). Interestingly, SRC4 contains an uncharacterized N‐terminal Domain (NTD, 1–41 amino acids, aa) and a typical TIR domain (42–190 aa), but it does not have typical NBS and LRR domains. In addition, SRC4 contains an uncharacterized domain which we named SNOR (Shortened NBS Oligomerization Region, 191–242 aa) as well as a Ca^2+^–binding structural domain EFh (EF‐hand, 243–305 aa), both of which were not reported in both typical and atypical NLR proteins previously (Figure 1B). These features identify SRC4 as an atypical R protein with a distinct domain architecture.

In contrast to SRC7‐TN (truncated TIR‐NBS form of SRC7), which displayed strong resistance to SMV, SRC4 showed only partial resistance to SMV (Figure 1C, left). EFh domain is broadly reported to bind Ca^2+^ and to be involved in Ca^2+^‐mediated cellular response (Wang, Z, et al., 2018). Therefore, we sprayed 10 mm CaCl_2_ on plant leaves. The SMV‐GFP assay showed that Ca^2+^ treatment significantly enhanced the antiviral activity of SRC4, whereas no detectable Ca^2+^‐dependent enhancement of SRC7‐TN activity was observed in this experiment (Figure 1C). Consistently, RT‐qPCR analysis showed that SRC4 reduced SMV CP RNA accumulation relative to EV, and Ca^2+^ treatment further reduced SMV CP RNA accumulation in SRC4‐expressing leaves. In contrast, SMV CP RNA accumulation remained at similarly low levels in SRC7‐TN‐expressing leaves with or without Ca^2+^ treatment (Figure 1E).

To further evaluate the role of Ca^2+^ in the antiviral activity of SRC4, we employed the Ca^2+^ channel inhibitors RR (ruthenium red) and GdCl_3_ (gadolinium chloride), alongside the Ca^2+^ chelators EGTA and EDTA. RR is known to block intracellular Ca^2+^ release by targeting ryanodine receptors, while GdCl_3_ inhibits extracellular Ca^2+^ influx by blocking plasma membrane Ca^2+^ channels (Lima et al., 2018). Both EGTA and EDTA act as competitive chelators by binding free Ca^2+^ and reducing intracellular Ca^2+^ availability. Notably, application of the inhibitors and chelators led to a significant reduction in SRC4‐mediated antiviral resistance (Figure 1D), indicating that the antiviral activity of SRC4 depends on the levels of endogenous Ca^2+^.

To assess the impact of different Ca^2+^ application methods on SRC4‐mediated resistance, we used three means: foliar spraying, supplementation in the culture soil, and watering with Ca^2+^ solution. In all cases, Ca^2+^ was provided either as CaCl_2_ or as Ca(NO_3_)_2_, and deionized water (ddH_2_O) served as the control treatment. The results demonstrate that the addition of exogenous Ca^2+^, regardless of the application methods, significantly enhanced SRC4‐mediated disease resistance (Figure 1F; Figure S1). Next, we examined the optimal Ca^2+^ concentration for maximal SRC4 activation by foliar spraying with CaCl_2_ solutions at various concentrations, where we found that 0.1% CaCl_2_ solution, equivalent to a Ca^2+^ concentration approximate 9.08 mM, was sufficient to enhance SRC4 activity (Figure S2).

Additionally, we investigated the effects of other common metal cations on SRC4‐mediated resistance by spraying the leaves with 10 mM solutions of MgCl_2_, CuSO_4_, ZnCl_2_, FeSO_4_, MnCl_2_, and KCl. Zn^2+^, Mn^2+^, and K^+^ did not show obvious effects, but both Mg^2+^ and Cu^2+^ significantly enhanced SRC4 antiviral activity. Surprisingly, Fe^2+^ severely inhibited SRC4 activity, indicating the distinct functions of these cations in activating SRC4 (Figure S3).

### *SRC4* expression levels are positively correlated with SMV resistance in soybean

**Figure 2:**
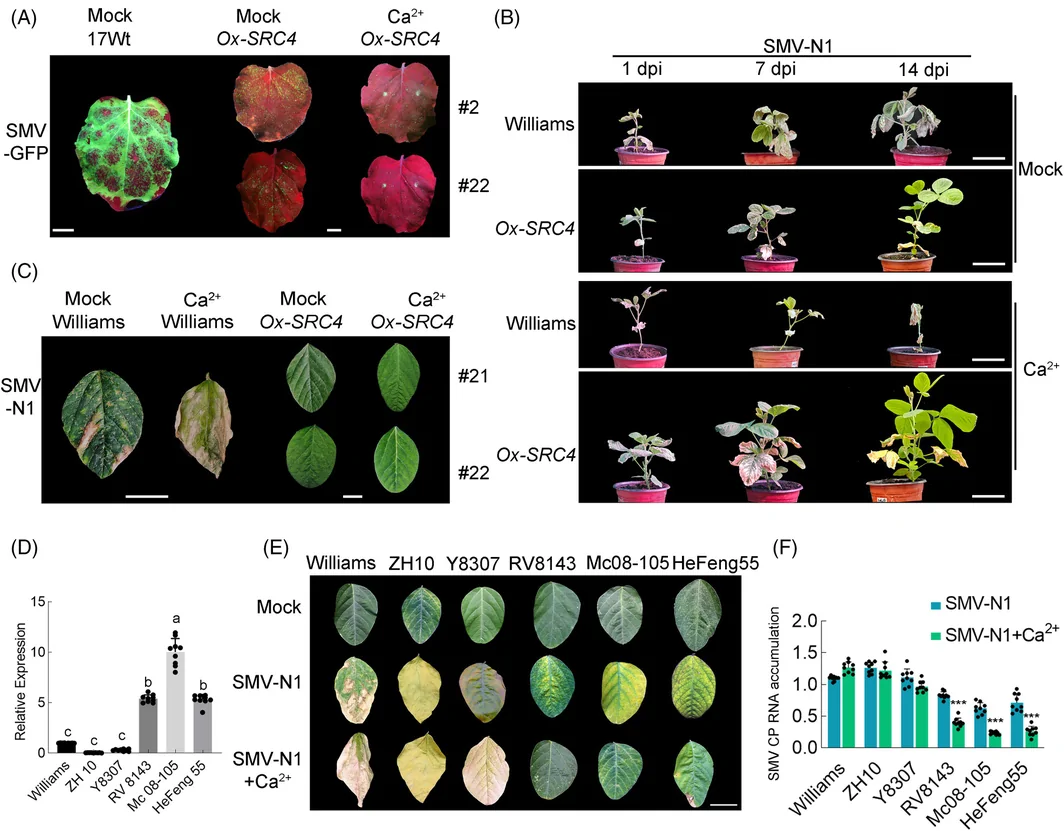
Disease symptoms of *SRC4*‐transgenic plants and different soybean varieties after SMV infection. (A) Ca^2+^ enhances SRC4 antiviral activity against SMV‐GFP. Both 17Wt and Ox‐*SRC4 N. benthamiana* (#2 and #22) plant leaves were infected with SMV‐GFP. Ca^2+^: 10 Mm CaCl_2_ was applied by foliar spraying. Mock: ddH_2_O. GFP was visualized under a handheld UV lamp (wavelength: 365 Nm) at 7 dpi. 17Wt: Wild‐type *N. benthamiana*; Ox‐*SRC4*: Transgenic *N. benthamiana* stably expressing *SRC4*, *n* ≥ 30. Scale bar: 1 Cm. (B, C) Virus inoculation and symptoms observation. Soybean plants at V2–V3 developmental stages were mechanically inoculated with SMV‐N1 by rubbing leaves with a homogenate derived from SMV‐infected tissues. The phenotype was observed at 1, 7, and 14 dpi. SMV‐N1: Sap inoculation with SMV‐N1. Ca^2+^: 10 Mm CaCl_2_ applied by foliar spraying. Mock: ddH_2_O. Williams: Wild‐type soybean cv. Williams 82; Ox‐*SRC4*: Transgenic Williams 82 stably expressing *SRC4*. Scale bar: 10 Cm. Representative leaves from two independent Ox‐*SRC4* soybean lines (#21 and #22) are shown in (C) at 20 dpi. Scale bar in (C): 1 Cm. (D) Steady‐state levels of *SRC4* mRNA in six soybean cultivars. Total RNA was extracted from leaf tissues at the V3 stage. *Actin* served as the internal control. Data are presented as mean ± SD. Statistical significance was determined by one‐way ANOVA followed by Tukey's multiple‐comparisons test. Different lowercase letters indicate significant differences at *P* < 0.05 (*n* = 3 biological replicates, with three technical replicates per biological replicate). (E) The disease symptoms of different soybean cultivars after SMV infection. Mock: ddH_2_O. The symptoms of leaves were observed at 35 dpi. SMV‐N1 + Ca^2+^: 10 Mm Ca^2+^ was applied via foliar spraying. Scale bar (B): 10 Cm. Scale bar (C): 1 Cm. (F) RT‐qPCR quantification of relative SMV CP RNA accumulation in systemic leaves after SMV‐N1 inoculation. Total RNA was extracted from a mixed sample of 10 leaves. Individual data points represent biological replicates. Data are presented as mean ± SD. Statistical differences were analyzed by one‐way ANOVA; *** indicates *P* < 0.001 (*n* = 3 biological replicates, each biological replicate included 3 technical replicates).

To precisely dissect the roles of SRC4, we generated SRC4 overexpression transgenic tobacco plants. The expression of SRC4 was confirmed by RT‐qPCR, gDNA PCR, and Western blot assays (Figure S4A–C). Ox‐*SRC4* tobacco showed enhanced resistance to SMV when compared with wild‐type tobacco. Notably, SRC4 activity was further enhanced by exogenous addition of Ca^2+^ on plants (Figure 2A). In addition, we also generated Ox‐*SRC4* soybean plants, and the transgenic plants were confirmed by RT‐qPCR, gDNA PCR, and Western blot assays (Figure S4D–F). Ox‐*SRC4* soybean was inoculated with the virus strain SMV‐N1 at 20 days post germination. Ox‐*SRC4* plants exhibited strong resistance against SMV‐N1 when compared to the control Williams plants that were susceptible to SMV infection. Although exogenous application of Ca^2+^ enhanced the survival of Ox‐*SRC4* plants, Ca^2+^ negatively influenced the growth of control Williams plants under SMV challenge (Figure 2B,C). Consistently, RT‐qPCR analysis showed that SMV CP RNA accumulation was lower in two independent Ox‐*SRC4* lines than in their respective wild‐type controls in both *N. benthamiana* and soybean, and Ca^2+^ treatment further reduced SMV CP RNA accumulation in the Ox‐*SRC4* lines (Figure S4G,H). These results indicate that SRC4 confers resistance to SMV in a Ca^2+^‐dependent manner.

Based on these observations, we further determined whether SRC4 contributes to the resistance to SMV in different soybean varieties. We first examined *SRC4* expression levels in different soybean varieties, in which we identified three low‐expression varieties, Williams, ZH10, and Y8307, and three high‐expression varieties, RV8143, Mc08‐105, and HeFeng55 (Figure 2D). The seedlings in V3 stage were infected with SMV‐N1 via sap inoculation. At 35 days postinoculation, disease symptoms were observed and the representative symptoms in systemic leaves were photographed. As shown in Figure 2E, three *SRC4* low‐expression varieties displayed strong disease symptoms, including systemic chlorosis and necrotic spots. By contrast, these symptoms were less severe in the three *SRC4* high‐expression varieties. Interestingly, when 10 mM CaCl_2_ was applied via foliar spraying, the disease symptoms were obviously ameliorated in three *SRC4* high‐expression varieties. However, the disease symptoms were not changed in the three *SRC4* low‐expression varieties (Figure 2E). RT‐qPCR analysis of SMV CP RNA accumulation in systemic leaves at 8 days postinoculation (dpi) showed that SMV CP RNA accumulation was 1.3‐ to 2.0‐fold higher in the three SRC4 low‐expression varieties than in the three SRC4 high‐expression varieties. Furthermore, exogenous Ca^2+^ application significantly decreased SMV CP RNA accumulation in the three SRC4 high‐expression varieties by 52–64%, but did not significantly reduce SMV CP RNA accumulation in the three *SRC4* low‐expression varieties (Figure 2F). Taken together, the above data demonstrated that the resistance strength against SMV in the investigated soybean cultivars was positively correlated with expression levels of *SRC4*, and exogenous application of Ca^2+^ improved the resistance to SMV.

### Validation of Ca^2+^ binding to EFh domain of SRC4

**Figure 3:**
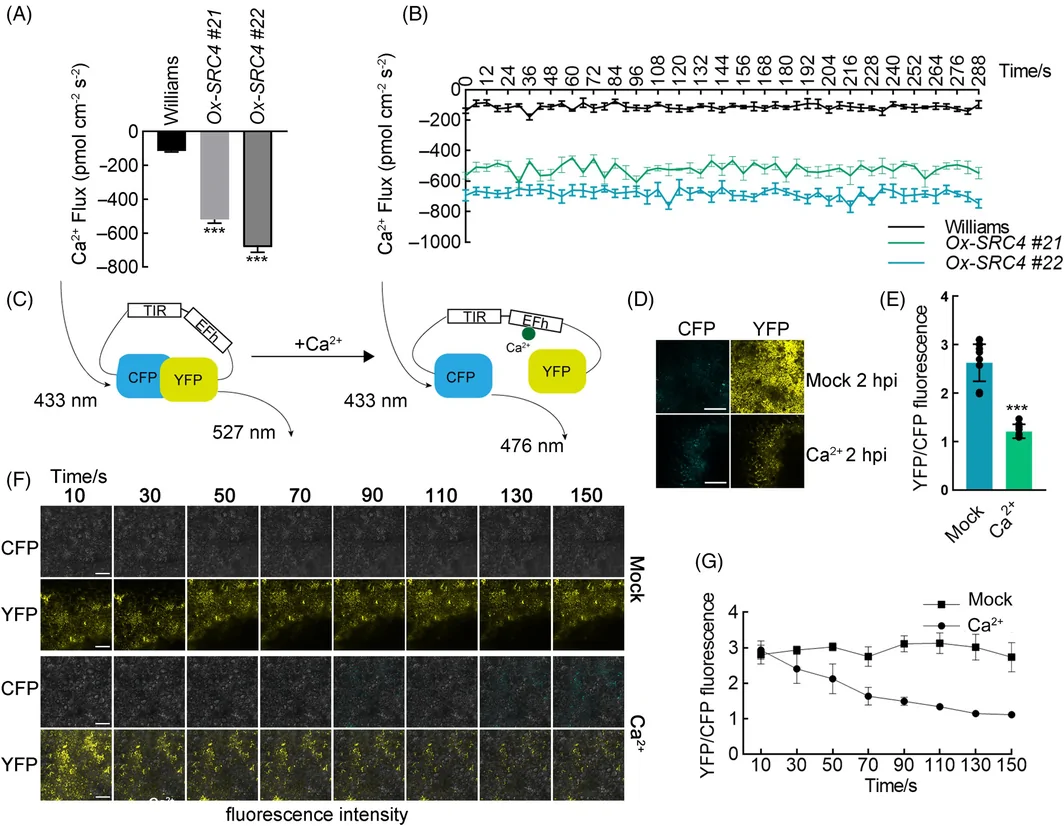
Ca^2+^ flux dynamics and Ca^2+^‐binding analysis for SRC4. (A) Non‐invasive microelectrode ion flux estimation (NMT) analysis of Ca^2+^ flux. Bar chart displays the Ca^2+^ flux rate in the xylem of soybean roots. Flow rate is valued in pico mol·cm^−2^·s^−1^, representing Ca^2+^ influx in the root. Data are presented as Means ± SD. Statistical differences were analyzed by one‐way ANOVA, *** indicates *P <* 0.001, *n* = 6. (B) Time‐course measurements of Ca^2+^ influx (pico mol·cm^−2^·s^−1^) dynamics in soybean. Ca^2+^ flux was measured at 12‐sec intervals for 288 sec. (C) Schematic representation of the Förster resonance energy transfer (FRET) construct. The SRC4 protein was fused with CFP at its N terminus and YFP at its C terminus to generate the CFP‐SRC4‐YFP FRET system. (D, E) Fluorescence intensity in CFP‐SRC4‐YFP. The construct was transiently expressed in *N. benthamiana* leaves. Detached leaves were treated with mock (1 × phosphate‐buffered saline) or Ca^2+^ (10 mM CaCl_2_) solution for 2 h. Fluorescence intensity was observed using a confocal microscope. (E) Box plot shows the ratio of YFP to CFP fluorescence. Data are presented as Means ± SD. Statistical differences were analyzed by one‐way ANOVA, *** indicates *P <* 0.001, *n* = 20. Scale bar: 50 μm. (F, G) YFP/CFP fluorescence ratio after the addition of Ca^2+^. The signals were recorded at different time points up to 150 sec. The numbers represent the time (s) following Ca^2+^ addition. Scale bar: 50 μm.

As SRC4 contains a Ca^2+^ binding EFh domain and exogenous application of Ca^2+^ enhances its antiviral activity, we asked whether SRC4 binds Ca^2+^. Interestingly, Ox‐*SRC4* plants exhibited a significant and stronger Ca^2+^ influx than Williams plants (Figure 3A). By measuring Ca^2+^ influx at different time points after SMV infection, Ox‐*SRC4* plants showed consistent and stronger Ca^2+^ influx than Williams plants (Figure 3B). This phenomenon resembles the Ca^2+^ signaling induced by NLR immune activation, suggesting that SRC4 participates in regulating Ca^2+^ flow in response to pathogen invasion. Notably, the NLR protein ZAR1 has been shown to trigger a rapid influx of Ca^2+^ upon recognition of specific pathogen effectors. However, despite the ability of RPS2 and RPS5 to recognize their respective effectors, their activation does not directly lead to a pronounced Ca^2+^ influx (Duxbury et al., 2016; Qi & Innes, 2013; Wang et al., 2019).

To investigate Ca^2+^‐induced conformational changes in SRC4, we generated an intramolecular FRET reporter by fusing CFP to the N terminus and YFP to the C terminus of SRC4 (CFP‐SRC4‐YFP; Figure 3C). Transient expression of this construct in *Nicotiana benthamiana* epidermal cells enabled ratiometric monitoring of Ca^2+^‐induced changes in intramolecular Förster resonance energy transfer (FRET). Two hours after exogenous Ca^2+^ application, confocal microscopy revealed a significant decrease in the YFP/CFP fluorescence ratio, indicating that Ca^2+^ addition may alter the distance between CFP and YFP (Figure 3D,E). This observation is consistent with FRET results in tracking Ca^2+^‐dependent protein structural rearrangements (Liese et al., 2023; Xu et al., 2024; Zhang et al., 2019). Upon the addition of Ca^2+^, a total of eight images were captured within 150 sec in 20‐sec intervals. During this period, a progressive increase in CFP fluorescence was observed, accompanied by a concomitant decrease in YFP fluorescence. This result indicates that, following Ca^2+^ addition, the CFP‐tagged N‐terminal region of SRC4 gradually separated from the YFP‐tagged C‐terminal region of SRC4 (Figure 3F). The fluorescence intensity ratio of CFP to YFP was used to monitor the dynamic changes in fluorescence. After addition of Ca^2+^, the fluorescence ratio of CFP‐SRC4‐YFP continuously decreased, with a noticeable slowing down of the decline rate at around 110 sec. In contrast, the mock group exhibited a stable fluorescence intensity ratio, indicating that the observed changes in the experimental group were specifically associated with the Ca^2+^‐induced conformational changes of SRC4 (Figure 3G).

### Characterization of the functional domains in SRC4

**Figure 4:**
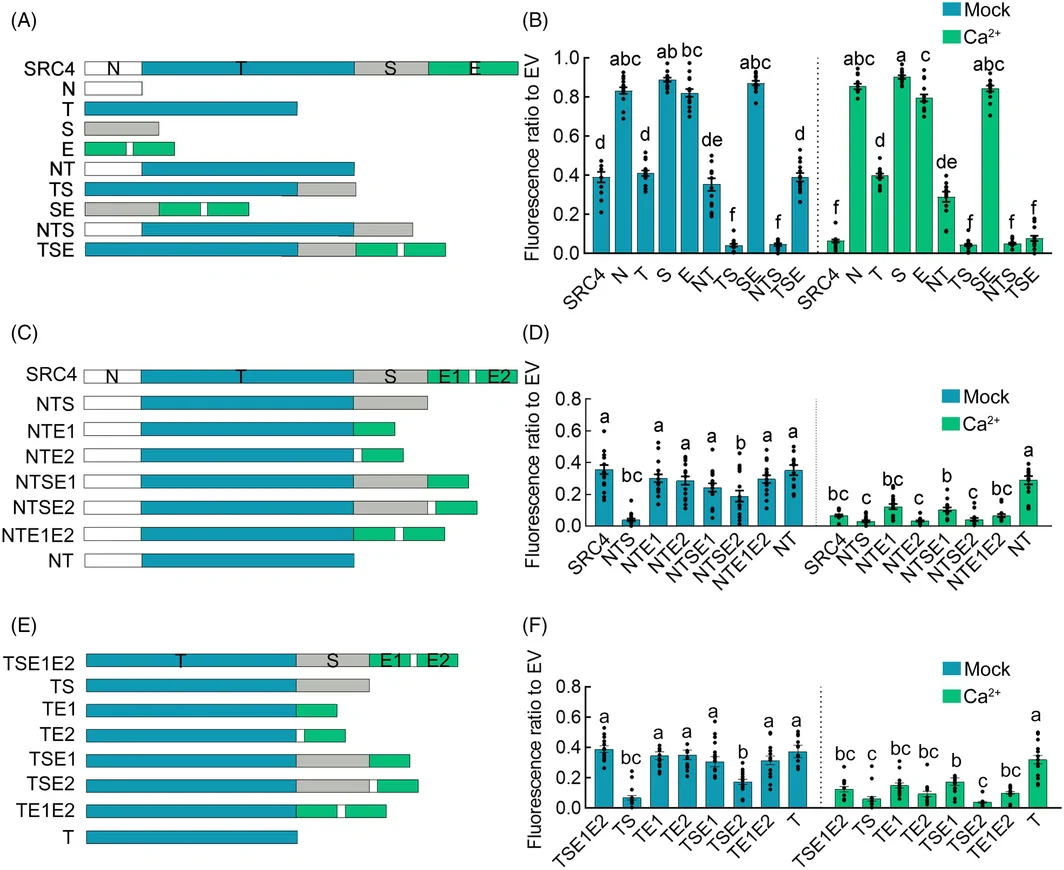
Truncation of SRC4 and antiviral activity assays. (A, C, E) SRC4 truncation schematic representation. N: NTD (1–41 aa); T: TIR (42–190 aa); S: SNOR (191–242 aa); E: EFh (243–305 aa); E1: EFh subdomain 1 (243–271 aa); E2: EFh subdomain 2 (272–305 aa). (B, D, F) Antiviral activity assays. The truncated proteins were transiently expressed in *N. benthamiana* and then the leaves were infected with SMV‐GFP. Ca^2+^ was applied with 10 mM CaCl_2_ on the leaves. Mock: ddH_2_O. GFP fluorescence was captured under a handheld UV lamp (wavelength: 365 nm) at 4 dpi and analyzed with Fiji software. The chart shows the fluorescence intensity ratio of experimental groups to EV group. Data are presented as Means ± SEM. Statistical significance was determined using Tukey's HSD test in SPSS 27.0. Different letters indicate significant differences at *P <* 0.05, *n* ≥ 30.

To illustrate the antiviral mechanism of SRC4, we truncated the protein based on the predicted domains. SRC4 contains a typical TIR domain (abbreviated as T, 42–190 aa) and three non‐typical domains, which are a NTD domain (abbreviated as N, 1–41 aa), a SNOR domain (abbreviated as S, 191–242 aa), and an EFh domain (abbreviated as E, 243–305 aa) that is comprised of two EFh7 subfamily Ca^2+^‐binding loops, which we designated as E1 (243–271 aa) and E2 (272–305 aa). In total, nine truncations were made (Figure 4A). The respective protein variants were transiently expressed in tobacco leaves and were determined for antiviral activity during SMV infection. TIR domain (T) and NT showed comparable antiviral activity to full‐length SRC4; however, the antiviral activity was completely abolished in N, S, E, and SE, indicating that the TIR domain confers basal resistance. Comparable activity of NT with TIR and SRC4 with TSE suggested that NTD does not influence the resistance of SRC4. Higher resistance of TS and NTS indicated that SNOR enhanced SRC4 antiviral activity. Lower resistance of SRC4 (full‐length NTSE) and TSE indicated that the EFh domain undermined the antiviral activity of SRC4. Notably, Ca^2+^ application did not activate NTD, SNOR, EFh, and SE in disease resistance, further supporting that NTD, SNOR, and EFh domains have no antiviral activity. By contrast, SRC4 and TSE, both carrying the EFh domain, showed enhanced resistance upon Ca^2+^ treatment, indicating that SRC4 positively responds to Ca^2+^ application via its EFh domain (Figure 4B; Figure S5A). RT‐qPCR analysis of SMV CP RNA accumulation further confirmed that the viral accumulation pattern of the truncation constructs was consistent with the GFP fluorescence phenotype (Figure S5B).

To investigate the regulatory target of EFh and dissect the function of two EFh subdomains, five extra truncation versions of SRC4, including the SNOR‐deleted NTE1E2, NTE1, NTE2, and EFh subdomain‐deleted NTSE1 and NTSE2 were generated (Figure 4C). Compared to NT, the antiviral activity of NTE1E2, NTE1, and NTE2 has no discernible changes. However, the antiviral activity of EFh‐containing NTSE1E2 (SRC4), NTSE1, and NTSE2 decreased significantly compared to that of NTS. Further examination showed that both E1 and E2 subdomains respond to Ca^2+^ application, and E2 displays higher activity than E1 (Figure 4D). Importantly, deletion of N‐terminal NTD domain does not influence the antiviral activity and Ca^2+^ responsiveness, supporting the conclusion that NTD is dispensable for SRC activity (Figure 4E,F).

We previously reported that the function of SRC7 relies on the nuclear oligomerization of both TIR and NBS domains to initiate immune signal transduction (Yan et al., 2022). As TIR domains in both SRC4 and SRC7 showed basal antiviral activity, we next investigated the role of SNOR and EFh domains of SRC4 by fusing these domains to TIR‐NBS or TIR domain of SRC7. Interestingly, the E2 subdomain slightly reduces SRC7's antiviral activity and the E1 dramatically decreases this activity. Both domains conferred Ca^2+^ positive responsiveness and disease resistance (Figure S6). In SRC7‐TIR background, the SNOR domain strengthened SRC7‐TIR activity, and EFh domains, either E1 and E2 combination or alone, reduced the resistance. Although the fusion of E1 or E2 subdomain enhanced antiviral activity of SRC7‐TIR after Ca^2+^ treatment, the activity is lower than that of SRC4 (Figure S7).

### The SNOR domain contributes to SRC4 self‐association and antiviral activity

Homology modeling reveals that SNOR domain (52 aa) locates at the N terminus of the NBS domain in dicotyledonous legumes and is often unannotated in plant genomes. Homologous sequences of the SNOR domain also appear in a small number of woody plants and are not found in monocotyledonous plants (Figure S8). Based on this sequence similarity and its contribution to SRC4 self‐association, we refer to this region as SNOR (shortened NBS oligomerization region).

**Figure 5:**
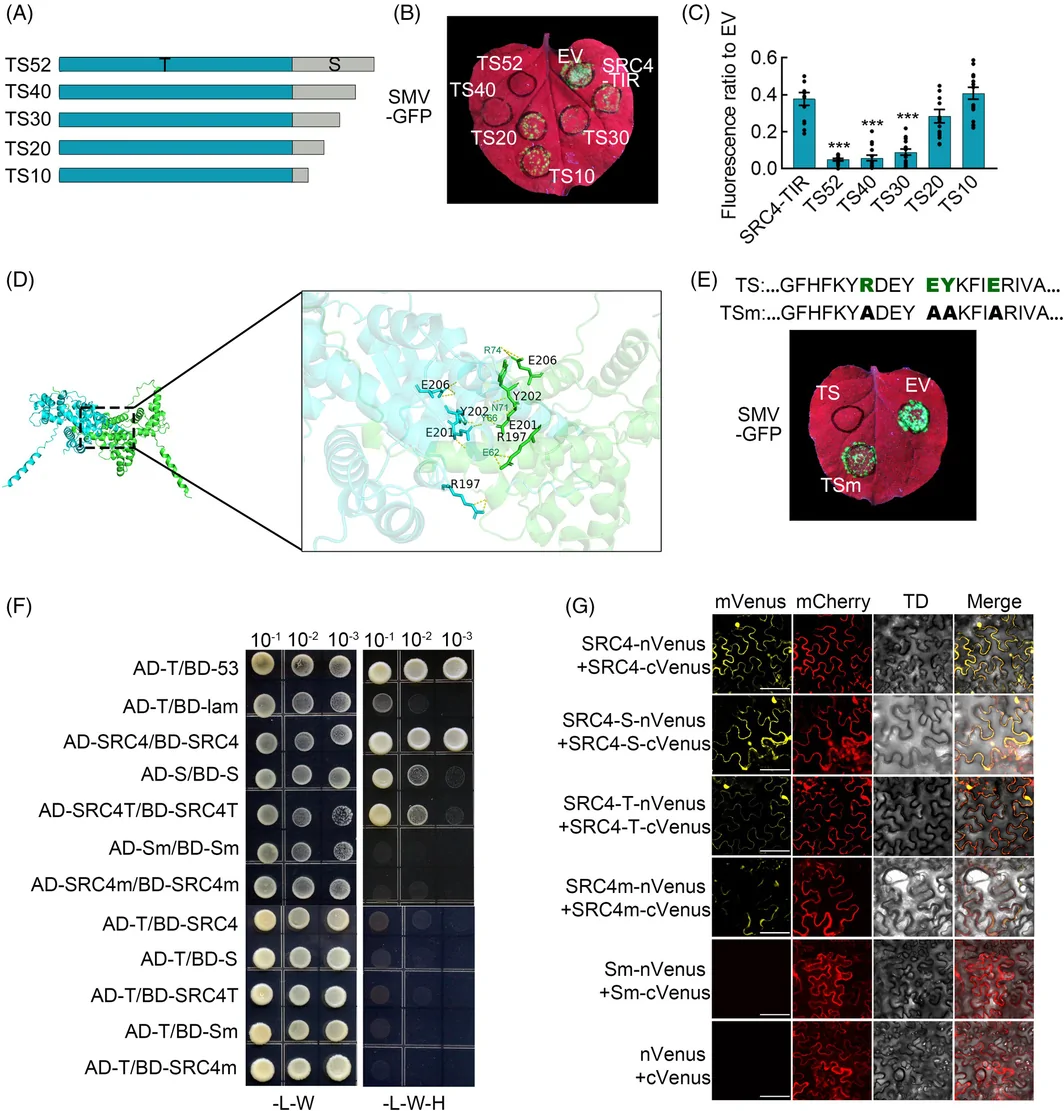
Role of the SNOR domain in SRC4 self‐association and antiviral activity. (A) Schematic of SNOR domain truncation. The truncations were generated at intervals of 10 aa from the C terminus within the TIR‐SNOR construct (TS52). (B, C) Antiviral activity assays for the truncated proteins. The proteins were transiently expressed in *N. benthamiana* leaves and then the leaves were infected with SMV‐GFP. GFP fluorescence was captured under a handheld UV lamp (wavelength: 365 nm) at 4 dpi and the representative pictures were shown in (B). GFP intensity was analyzed with Fiji software and the chart (C) shows the fluorescence intensity ratio of experimental groups to EV group. Data are presented as mean ± SEM. Statistical differences were analyzed by one‐way ANOVA, *** means *P <* 0.001, *n* ≥ 30. (D) AlphaFold 3 model of a possible intermolecular TIR–SNOR interface. R197, E201, Y202, and E206 are located within the region designated as SNOR in this study, whereas E62, Y66, N71, and R74 are located in the TIR region of the other SRC4 molecule. Dashed lines indicate model‐predicted polar contacts: R197–E62, E201–Y66, E201–N71, Y202–N71, and E206–R74. The top‐ranked Model 0 had an ipTM value of 0.47. These contacts are model predictions and do not represent experimentally resolved hydrogen bonds. (E) Effects of residue substitutions on TS antiviral activity. The interface‐associated residues R197, E201, Y202, and E206 are shown in green. TSm: mutant TS construct in which R197, E201, Y202, and E206 were replaced with alanine. Antiviral activity assays were performed as described in (B), and a representative result is shown. (F) Yeast two‐hybrid analysis for SRC4 self‐association. Truncated and mutant SRC4 variants were examined for the interaction assays. Sm: the same alanine substitution at key residues in SNOR domain indicated in (E); T: SRC4‐TIR domain; SRC4m: full‐length SRC4 with SNOR domain mutated to Sm. Controls: pGADT7‐T + pGBKT7‐53 (positive), pGADT7‐T + pGBKT7‐lam (negative). The SRC4‐TIR construct is designated as BD‐SRC4T to distinguish from AD‐T controls. (G) BiFC assays for SRC4 self‐association. nVenus+cVenus served as a negative control. Two days after inoculation, Venus signals were observed. mCherry: Histone3‐mCherry for labeling nucleus; LTI6b‐mCherry for labeling plasma membrane. Scale bar: 50 μm.

To investigate the role of the SNOR domain, it was truncated from the C terminus at intervals of 10 aa in TIR‐SNOR (TS52), leading to four truncated proteins, TS40, TS30, TS20, and TS10 (Figure 5A). TS40 and TS30 showed comparable activities to TS52, but TS20 and TS10 almost cannot enhance the resistance of the TIR domain (Figure 5B,C), demonstrating that the 30 aa at the N terminus of the SNOR domain are sufficient to enhance TIR‐mediated resistance.

AlphaFold 3 modeling of a TS (TIR–SNOR) dimer suggested a possible intermolecular interface between the region designated as SNOR in one TS molecule and the TIR region of the other molecule. The model indicated several possible polar contacts across this interface, including R197‐E62, E201‐Y66, E201‐N71, Y202‐N71, and E206‐R74 (Figure 5D). When R197, E201, Y202, and E206 were replaced with alanine, the resulting TSm construct (R197A, E201A, Y202A, and E206A) showed significantly compromised antiviral activity (Figure 5E). In yeast two‐hybrid (Y2H) assays, full‐length SRC4 showed self‐interaction. The isolated SNOR and TIR regions also showed self‐interaction. When the four interface‐associated residues were mutated in full‐length SRC4 (SRC4m) or in the SNOR region (Sm), self‐interaction was no longer detected in the Y2H assay (Figure 5F). To further examine this observation, we performed a BiFC assay. Both the isolated TIR and SNOR regions produced Venus fluorescence signals, although the signal from the TIR region was substantially weaker than that from full‐length SRC4. No interaction signal was observed for Sm, whereas a weak signal remained for SRC4m (Figure 5G). These results suggest that the region designated as SNOR contributes to SRC4 self‐association and TIR‐dependent antiviral activity, although the precise structural basis of this interaction remains unresolved.

### Effects of different EF‐hand domains on Ca^2+^‐mediated resistance

**Figure 6:**
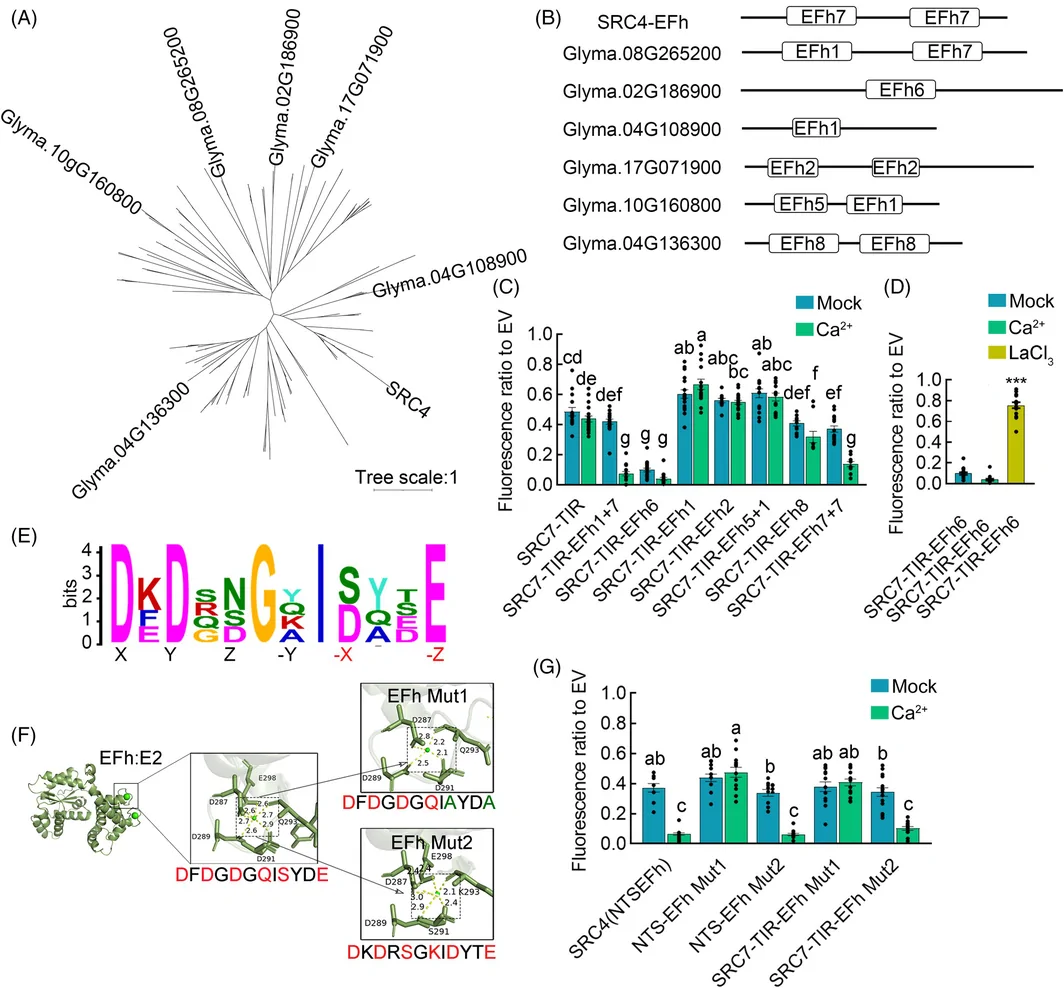
Identification of the EFh subdomains with Ca^2+^‐responsive antiviral activity. (A) Phylogenetic analysis of 202 EFh family genes in soybean. Representative members in each subfamily are shown. (B) Seven representative EFh domain structures. Six different EFh subdomains, the EFh1, EFh2, EFh5, EFh6, EFh7, and EFh8, are shown. In SRC4‐EFh, EFh7‐EFh7 corresponding to the E1‐E2 structure was shown in Figure 1B. (C) Involvement of EFh domains in Ca^2+^ responsiveness and TIR‐mediated antiviral activity. Seven representative EFh domains shown in (B) were fused in‐frame after SRC7‐TIR domain. Labels such as EFh1 + 7 indicate the subfamily identities of the two Ca^2+^‐binding loops naturally present in the tested soybean EFh domain; each intact EFh domain was fused in‐frame downstream of the SRC7 TIR domain. GFP intensity was analyzed with Fiji software and the chart shows the fluorescence intensity ratio of experimental groups to EV group. Data are presented as mean ± SEM. Statistical significance was determined using Tukey's HSD test in SPSS 27.0. Different letters indicate significant differences at *P <* 0.05, *n* ≥ 15. (D) Fluorescence in SRC7‐TIR‐EFh6. The experiments were performed as described in (C). Statistical differences were analyzed by one‐way ANOVA, *** indicates *P <* 0.001, *n* ≥ 15. (E) Comparison of 12‐amino acid Ca^2+^‐binding motif in the EFh domains encoded by the seven genes in (B). Six key residues (X, Y, Z, ‐Y, −X, and ‐Z) coordinate Ca^2+^ in a pentagonal bipyramidal geometry at Positions 1, 3, 5, 7, 9, and 12. The invariant Glu at Position 12 (yellow) provides two oxygens for coordinating Ca^2+^. (F) Mutations in the 12‐amino‐acid Ca^2+^‐binding loop of SRC4‐EFh E2. Structures of the original and mutant motifs were predicted by AlphaFold 3. The numbers in the dashed boxes indicate the model‐predicted distances between Ca^2+^ and the coordinating residues. The red letters denote the X, Y, Z, ‐X, ‐Y, and ‐Z key sites. EFh Mut1 (Mutation 1): the critical binding sites, ‐X and ‐Z, were mutated to alanine (A, green). EFh Mut2 (Mutation 2): the 12 aa Ca^2+^‐binding motif in SRC4‐EFh E2 was replaced by its equivalent from EFh6 domain. (G) Effects of mutations in EFh on Ca^2+^‐responsiveness and antiviral activity. The experiments were performed as described in (C). NTS‐EFh Mut is the EFh domain mutated and linked to the NTS domain of SRC4, and SRC7‐TIR‐EFh Mut is the EFh domain mutated and linked to the TIR domain of SRC7. Statistical significance was determined using Tukey's HSD test in SPSS 27.0. Different letters indicate significant differences at *P <* 0.05, *n* ≥ 30.

Through domain prediction, we identified a total of 202 genes encoding EF‐hand (EFh) domains in soybean (Figure 6A). These EFh domains are classified into six subfamilies: EFh1, EFh2, EFh5, EFh6, EFh7, and EFh8. To distinguish the roles of different EFh domains in modulating TIR‐mediated resistance, EFh domain‐encoding sequences were cloned from six representative subfamilies (Figure 6B). Because the SRC7 TIR domain represents a relatively typical and previously well‐characterized antiviral TIR module (Yan et al., 2022), it was selected as a common recipient for these EFh domains. The resulting artificial TIR‐EFh fusion proteins provided a simplified SRC4‐like architecture, allowing the effects of different EFh subfamilies on TIR‐mediated antiviral activity and Ca^2+^ responsiveness to be compared in the same background. Their activities against SMV‐GFP were subsequently examined using the transient expression system. EFh1, EFh2 + 2, and EFh5 + 1 have no effects on disease resistance, but EFh8 + 8 and EFh1 + 7 improved resistance upon Ca^2+^ treatment although they did not influence basal activity. In particular, EFh1 + 7 displays a stronger Ca^2+^ response than EFh7 + 7 from SRC4. Interestingly, EFh6 significantly enhanced the antiviral activity of SRC7‐TIR domain both in the presence and in the absence of exogenous Ca^2+^ (Figure 6C). The resistance of TIR + EFh6 is inhibited by LaCl_3_, indicating that the role of EFh6 in enhancing resistance is still related to its response to Ca^2+^, but with a higher sensitivity to cytosolic Ca^2+^ concentration (Figure 6D). These results suggest that Ca^2+^ cannot activate all EFh domains‐mediated resistance, and EFh domains show functional divergence which was likely attributed to their specific Ca^2+^ binding motifs.

The Ca^2+^‐binding properties of EFh domains are mediated by a highly conserved 12‐amino‐acid loop, in which six residues at positions X, Y, Z, −Y, −X, and −Z coordinate Ca^2+^ in a pentagonal bipyramidal geometry. We therefore compared the conservation of these motifs among EFh domains (Figure 6E). Because the −X and −Z positions are important for Ca^2+^ coordination and E2 was identified as the major Ca^2+^‐responsive motif of SRC4, both residues in the SRC4 E2 loop were substituted with alanine to generate EFh Mut1 (Figure 6F). EFh Mut1 was fused to SRC7‐TIR and tested for antiviral activity against SMV‐GFP in *N. benthamiana*. The substitutions completely abolished Ca^2+^‐dependent antiviral enhancement, supporting the role of the E2 loop in Ca^2+^ responsiveness (Figure S9). Because EFh6 showed a particularly strong and distinct enhancement of TIR‐mediated antiviral activity among the EFh domains examined, we selected EFh6 as the donor for EFh Mut2. The 12‐amino‐acid loop of SRC4 E2 was replaced with the corresponding EFh6 sequence to test whether this loop accounted for the distinctive activity of EFh6. EFh Mut2 conferred Ca^2+^‐responsive antiviral enhancement on both SRC7‐TIR and SRC4‐NTS but did not reproduce the EFh6‐mediated enhancement of basal antiviral activity in the absence of exogenous Ca^2+^. These results indicate that the 12‐amino‐acid Ca^2+^‐binding loop contributes to Ca^2+^ responsiveness but is insufficient to confer the full antiviral‐enhancing activity of the intact EFh6 domain, which may additionally depend on other sequence or structural features (Figure 6G).

### SRC4 interacts with coat protein of SMV via EFh domain

**Figure 7:**
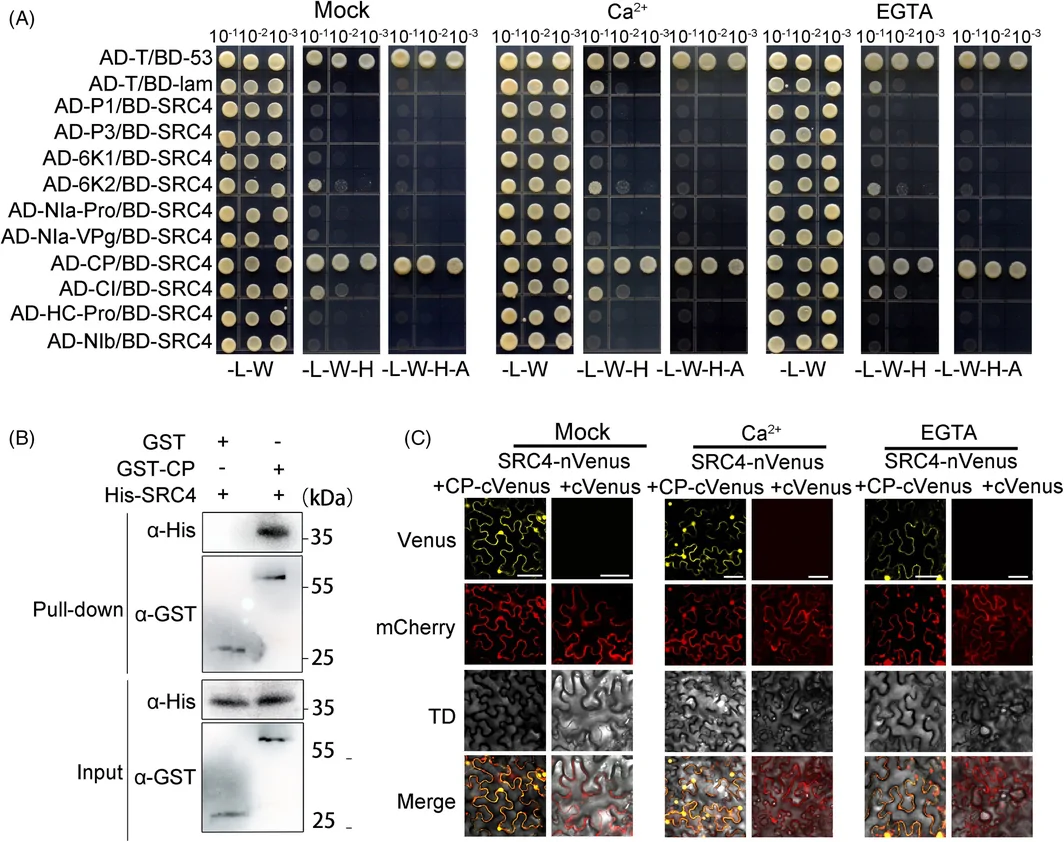
Interaction of SRC4 with SMV‐CP. (A) SRC4 interacts with SMV‐CP in yeast two‐hybrid assays. The Ca^2+^ and EGTA were added as 10 mM CaCl_2_ and 10 mM EGTA, respectively. Mock: ddH_2_O. pGADT7‐T + pGBKT7‐53 is the positive control and pGADT7‐T + pGBKT7‐lam is the negative control. (B) SRC4 interacts with SMV‐CP in GST pull‐down assays. GST‐CP fusion protein was expressed in *E. coli* BL21 and purified using glutathione‐Sepharose beads. *E. coli* lysate with His‐tagged SRC4 recombinant protein was incubated with the immobilized GST‐CP to capture interacting complexes. (C) SRC4 interacts with SMV‐CP in BiFC assays. SRC4‐nVenus and CP‐cVenus were co‐expressed in *N. benthamiana* by transient expression system. The Ca^2+^ and EGTA were added as 10 mM CaCl_2_ and 10 mM EGTA, respectively. SRC4‐nVenus+cVenus served as a negative control. At 2 days post infiltration, Venus and mCherry signals were recorded. mCherry: Histone3‐mCherry for labeling cell nucleus and LTI6b‐mCherry for labeling plasma membrane. Scale bar: 50 μm. CP: SMV coat protein (CP).

NLR receptors play a crucial role in plant immunity by directly or indirectly recognizing pathogen effectors. To investigate the mechanism of SRC4‐mediated resistance, we examined the interactions of SRC4 with the 10 canonical mature proteins produced from the SMV polyprotein: P1, HC‐Pro, P3, 6 K1, CI, 6 K2, NIa‐VPg, NIa‐Pro, NIb, and CP (Yan et al., 2022). The small trans‐frame protein P3N‐PIPO was not examined separately in this assay. In Y2H assays, SRC4 interacted with CP, whereas no interaction was detected with the other nine tested mature proteins. The impact of Ca^2+^ on the interaction was assessed by exogenous addition of 10 mM Ca^2+^ or EGTA. The result showed that Ca^2+^ did not influence SRC4‐CP interaction (Figure 7A). The interaction was further confirmed by pull‐down assays, in which GST‐CP successfully immunoprecipitated His‐SRC4 while GST alone could not (Figure 7B).

We also determined the subcellular localization of SRC4 and the roles of its functional domains. As shown in Figure S10, SRC4 localized at both the plasma membrane and the nucleus, and deletion of the SNOR and EFh domains did not affect this localization pattern, suggesting that the N‐terminal NTD‐TIR region contributes to the plasma‐membrane localization of SRC4. By contrast, the EFh domain alone localized to the nucleus without an evident plasma‐membrane signal. In BiFC assays, SRC4‐nVenus showed interaction with CP‐cVenus and the Venus signal overlapped with membrane‐localized mCherry signal, indicating that they interacted at the plasma membrane, and addition of Ca^2+^ and EGTA did not alter the interactions (Figure 7C).

Next, we determined which domain of SRC4 is responsible for the interaction with CP. We generated three SRC4 truncations from its C terminus and examined the interactions with CP. Deletion of E2 (272–305 aa) did not affect the interaction, while further deletion of E1 (272–276 aa) abolished the interaction, indicating that E1 is indispensable for SRC4's interaction with CP. In addition, the EFh domain alone is fused with nVenus to complement with CP‐cVenus. It showed that the EFh domain and CP exhibited a strong interaction at the cell membrane (Figure S11). The above result indicated that SMV recognition by SRC4 is mediated via interaction with CP and that the EFh domain is necessary and sufficient for this interaction.

### SRC4 improves soybean plant growth

**Figure 8:**
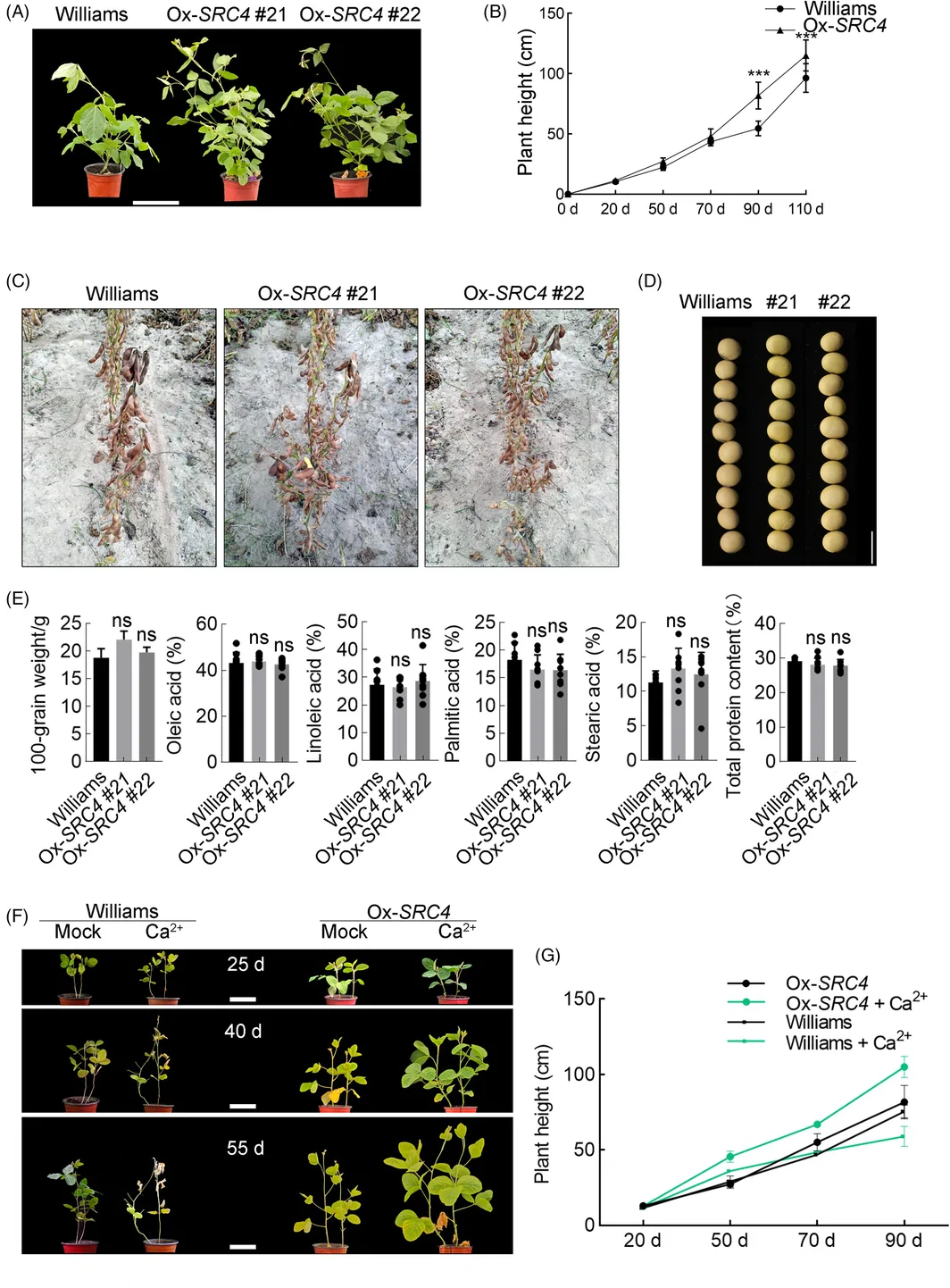
Overexpression of *SRC4* improves soybean plant growth. (A) Growth phenotype of Ox‐*SRC4* soybean plants. The plants were grown in the greenhouse under a 10‐h photoperiod. Representative phenotypes of two independent Ox‐*SRC4* lines (#21 and #22) are shown at 90 days after germination. Scale bar: 15 cm. (B) Plant height of Ox‐*SRC4* plants. Growth conditions were the same as in (A). Plant height was measured at the indicated time points. Ox‐*SRC4* data were pooled from two independent lines (#21 and #22). Data are presented as Means ± SD, *n* = 7 (biological replicates). Statistical differences were analyzed by one‐way ANOVA, *** indicates *P <* 0.001. (C) Growth phenotype of Ox‐*SRC4* plants at the pod‐setting stage. (D) Phenotype of Ox‐*SRC4* seeds. Scale bar: 1 cm. (E) Seed quality traits. Data are presented as mean ± SD. ns indicates no significant difference. Statistical differences were analyzed by one‐way ANOVA. Groups without asterisks showed no significant differences (*n* = 9 biological replicates). (F) Supplementation with Ca^2+^ facilitates Ox‐*SRC4* plant growth. After germination, the seedlings were treated with Ca^2+^ (50 mM CaCl_2_). Photographs were taken at 25, 40, and 55 days after germination. The plants were grown in a greenhouse under the same conditions as (A). Scale bar: 10 cm. (G) Height of Ox‐*SRC4* plants upon Ca^2+^ treatment. The plant height was measured at 20, 50, 70, and 90 days after germination.

Under greenhouse conditions, we monitored the growth of Ox‐*SRC4* soybean and observed that during the first 70 days post germination, its growth rate was comparable to Williams, the control plants. However, when the plants approached 90 days post germination and transited to the flowering and pod‐setting stages, Ox‐*SRC4* soybean exhibited a significantly greater plant height than Williams (Figure 8A,B; Figure S12A).

In the field trials conducted at Nanbin Farm, Hainan (19°32′ N, 109°12′E; elevation 18 m), we observed no significant differences in plant growth between Ox‐*SRC4* lines and the control during the vegetative stage and the pod‐setting stage (around 90 days, Figure S12B). Additionally, Ox‐*SRC4* soybean plants exhibited no notable differences in pod number or yield compared to Williams (Figure 8C). Furthermore, key agronomic traits, including 100‐seed weight, oleic acid, linoleic acid, stearic acid, and palmitic acid content, remained unaffected (Figure 8D,E). These findings suggest that overexpression of *SRC4* does not compromise plant growth or yield, although the plants gain improved disease resistance.

Because Ox‐*SRC4* soybean exhibited normal growth, we further evaluated their response to high concentration of Ca^2+^ (50 mM). At 25 days after germination, a high concentration of exogenous Ca^2+^ was applied. By Day 40, although both Ox‐*SRC4* plants and Williams showed accelerated growth under Ca^2+^ treatment compared to the mock group, Williams exhibited obvious leaf abscission, indicating a sensitivity to elevated Ca^2+^ levels. In contrast, Ox‐*SRC4* plants maintained healthy growth without visible stress symptoms. By Day 55, Ox‐*SRC4* plants under Ca^2+^ treatment exhibited increased plant height and expanded leaf area compared to Williams. These results show that overexpression of *SRC4* enhances plant tolerance to high‐concentration Ca^2+^ stress. Surprisingly, it also promotes plant growth under high‐Ca^2+^ conditions (Figure 8F,G). In addition, with Ca^2+^ supplementation, there was no significant growth loss but the antiviral capacity was significantly enhanced (Figure S13).

## DISCUSSION

In this study, we revealed a novel Ca^2+^ involved disease resistance mechanism against SMV in soybean. SRC4 is an atypical NLR protein containing both a TIR domain and a Ca^2+^‐binding EF‐hand (EFh) domain. Unlike typical NLR proteins, which rely on complex signaling networks associated with plasma membrane (PM) Ca^2+^ channels (Bi et al., 2021; Jacob et al., 2021), SRC4 not only facilitates rapid Ca^2+^ influx but also directly binds to Ca^2+^ to enhance resistance, providing a new strategy for immune activation.

In plants, the TIR domain plays a pivotal role in immunity, particularly in the context of NLR proteins (Horsefield et al., 2019; Wan et al., 2019). *Arabidopsis thaliana* TIR‐only protein RBA1 has been shown to mediate pathogen detection and regulate cell death independent of NBS and LRR domains, which are essential for the function of many NLR proteins. RBA1‐induced cell death relies on its oligomerization, which is facilitated by TIR‐TIR dimerization (Nishimura et al., 2017; Zhang et al., 2017). Here, we found that the TIR domain of SRC4 acts similar as that of RBA1, with the TIR domain alone being capable of conferring basal resistance. Our previous work also showed that TIR domain in boosting plant immunity was exemplified by dimerization in SRC7. Moreover, TNL proteins such as ROQ1 can assemble into tetrameric resistosomes structured as dimers of dimers, expanding the oligomerization modes beyond simple TIR‐mediated dimerization (Martin et al., 2020). In addition, the 52‐aa region designated as SNOR enhanced TIR‐mediated antiviral activity and contributed to SRC4 self‐association. Sequence comparison showed that homologous regions occur at the N‐terminal portion of NBS domains in dicotyledonous plants, particularly in legumes, suggesting that SNOR may represent a shortened NBS‐related sequence. The AlphaFold 3 model suggested a possible intermolecular TIR‐SNOR interface in the TS dimer, but the precise structural arrangement of this interface remains unresolved. Taken together, these results suggest that the SNOR region contributes to SRC4 self‐association and immune activity, although its precise structural relationship to typical TIR and NBS domains remains to be determined.

In typical NLR proteins, the LRR domain maintains NLR at rest state, and deletion of the LRR domains in *Arabidopsis* NLR proteins RPS2, RPS5, and RPP1A induce HR in the plants (Ma et al., 2020). LRR is required for effector recognition, for example, the effector of the parasitic downy mildew ATR1 can be specifically recognized by the LRR domain of RPP1 to generate a defense response (Goritschnig et al., 2016; Krasileva et al., 2010). Mutations in these regions can alter the recognition specificity. In our study, the EFh domain of SRC4 serves as a pathogen recognition domain, and it can significantly inhibit the resistance of the TIR‐NBS. We confirmed that EFh interacts with SMV‐CP. CP is also an important target for the recognition of NLR‐like resistance proteins in plants, playing an “effector” role in TIR‐NLR‐mediated immune activation (Bonnamy et al., 2023; Grech‐Baran et al., 2021).

With increasing genomic data available, many atypical NLRs carrying integrated domains (NLR‐IDs) have been identified (Sarris et al., 2016). The EFh domain is one of the most versatile Ca^2+^‐binding motifs in plants. In canonical Ca^2+^ signaling, CaMs and calmodulin‐like proteins (CMLs) contain four EFh domains. Upon Ca^2+^ binding, their conformation is changed to modulate downstream immune and stress responses. CBLs possess three EFh motifs and form CBL‐CIPK kinase complexes, which are the key modules in osmotic and ion stress responses. Such functional asymmetry provides an evolutionary blueprint for integrated domains (IDs) in atypical NLR immune receptors. EFh, like WRKY or HMA IDs, may retain only a subset of their ancestral functions, such as Ca^2+^‐induced conformational switching. Structural analyses of other EF‐hand proteins, such as calmodulin, suggest that Ca^2+^ binding induces a “closed‐to‐open” transition (Shimoyama & Takeda‐Shitaka, 2017). Our results suggest that Ca^2+^ perception by the SRC4 EFh domain contributes to the modulation of SRC4‐mediated resistance output. In our study, the SRC4 EFh domains modulate its resistance by attenuating the enhanced activity of the adjacent SNOR domain, suggesting the important role of integrated domains.

The subcellular localization of NLR proteins essentially influences their ability to recognize pathogen effectors and activate downstream immune responses (Bernoux et al., 2023). The studies on rice NLR proteins, such as OsRac1 and OsRac4, highlight the essential roles of their subcellular localization for disease resistance (Chen et al., 2010). In our study, SRC4 is localized at the cell membrane and nucleus. This localization may define SRC4's ability to recognize CP proteins at the plasma membrane to initiate resistance and is responsible for the enhanced influx of Ca^2+^ in Ox‐*SRC4* soybean. Although Ca^2+^ signaling is essential for plant immune responses, excessive or prolonged activation of immune pathways can have detrimental effects on plant growth. Therefore, plants must adequately control the intensity and duration of calcium signals to ensure that immunity is promptly activated but does not interfere with plant growth and development (Singh & Pandey, 2020). In this study, we observed that although calcium‐induced activation of SRC4 enhanced antiviral activity, the growth of plants under controlled calcium concentrations remained unaffected. We speculate that this could be attributed to the intrinsic properties of Ca^2+^.

It has been shown that calcium signaling pathways in some cases have a distinct regulatory role. This mechanism ensures that immunity is activated only when necessary and avoids detrimental effects on plant growth (Wang & Luan, 2024; Yuan et al., 2017). Although Ca^2+^ has been shown to play a critical role in plant immunity, it is also a vital secondary messenger involved in various cellular processes, including plant growth and development. Ca^2+^ acts as co‐signals in many cellular events, and the EF‐hand domain is key to transforming calcium signals into functional responses. For example, calcium gradients and oscillations are essential for many physiological processes, such as pollen tube elongation, root hair formation, and cell division (Brost et al., 2019; Qu et al., 2016). The ability of EF‐hand‐containing proteins to regulate these calcium signals is crucial for maintaining proper cellular function during the developmental stages. For instance, in root hair formation, EF‐hand proteins regulate calcium influx through CNGC channels, which is necessary for maintaining the growth and polarity of root hairs (Fang et al., 2024).

Ca^2+^ signaling in plant immune responses is tightly regulated, which not only ensures effective immune responses but also prevents cell damage due to excessive Ca^2+^ accumulation. In order to inhibit the effect of excessive accumulation of Ca^2+^ on plant growth, certain Ca^2+^ binding proteins can help alleviate toxicity under high‐Ca^2+^ environments by sequestering excess Ca^2+^ in cell compartments (Yáñez et al., 2020; Yang et al., 2007). CDPKs, such as CaCDPK1, have an EF‐hand motif, which allows them to bind Ca^2+^ and regulate a variety of cellular functions, including attenuating the accumulation of excess Ca^2+^ (Dekomah et al., 2022; Yip Delormel & Boudsocq, 2019). Our study demonstrates that the regulation of Ca^2+^ by SRC4 not only enhances immune responses but also alleviates the toxicity of excessive Ca^2+^. This phenomenon suggests that although Ca^2+^ was accumulated in plants at a higher level, SRC4 could tolerate high Ca^2+^ environments by binding the intracellular Ca^2+^. SRC4's EFh domain likely participates in maintaining intracellular Ca^2+^ balance and protects plants from the negative effects of excessive Ca^2+^.

The dual role of Ca^2+^ signaling in immunity and growth may help explain why EF‐hand proteins are widely distributed in plants. They not only serve as essential regulators of immune responses but also fine‐tune the complex network of signaling pathways required for proper growth and development (Chen et al., 2015; Mohanta et al., 2019). The ability to integrate both defense and growth functions through a shared signaling pathway suggests that EF‐hand proteins are key modulators of plant health, capable of coordinating multiple processes of plant physiology (Gao et al., 2014; Zeng et al., 2017). In Ox‐*SRC4* soybean, the addition of exogenous Ca^2+^ not only promoted plant growth but also enhanced antiviral responses. It indicates that *SRC4* can regulate immune activation while avoiding an imbalance between growth and resistance. Interestingly, we showed that the basal expression level of SRC4 was higher than that of other typical R genes under unchallenged conditions (Zhou et al., 2025), indicating that SRC4 is involved in growth regulation in addition to its role in immune responses. Furthermore, the addition of exogenous Ca^2+^ did not affect soybean agronomic traits. The balance of plant defense and growth is an important topic. The gene pairs, such as PigmR‐PigmS, are reported to balance this trade‐off in rice (Deng et al., 2017). Similarly, a single transcription factor, such as IPA1, was also reported to balance this trade‐off in a phosphorylation‐dependent manner (Jing Wang, Z, et al., 2018). However, the gene such as SRC4, which directly senses Ca^2+^ to regulate disease resistance and achieves a balance between immune response and yield, is barely reported.

In conclusion, SRC4, a Ca^2+^‐regulated atypical NLR protein, plays a significant role in enhancing plant resistance while maintaining balanced growth in soybean plants. Our study highlights the importance of NLR EFh of SRC4 in strengthening immunity by sensing Ca^2+^ levels in plants. Future research will explore the role of SRC4 in Ca^2+^ signaling regulation and investigate how to achieve the optimal balance between resistance and yield under field conditions. Nevertheless, exogenous Ca^2+^ supplementation could serve as an effective strategy to enhance plant immunity and to optimize crop yield in *SRC4* overexpressing plants.

## EXPERIMENTAL PROCEDURES

### Sequence analysis, gene cloning, and protein interaction

Candidate gene sequences were obtained from the soybean (*Glycine max*) genome database (Wm82.a2.v1, https://soybase.org/) for soybean cultivar Williams82. Gene cloning, truncation constructs, Y2H, and BiFC analysis were carried out by the methods described previously (Yan et al., 2019). SRC4 dimer models were generated using AlphaFold 3, and the top‐ranked model was used to examine candidate interface‐associated residues.

### Plant materials and growth conditions

All plant materials were grown in a standard greenhouse at 22°C–25°C with a photoperiod of 14:10 (light: dark) and relative humidity of 60–70%. Light intensity was maintained at 150–200 μmol photons m^−2^·s^−1^ using LED panels. Multiple soybean cultivars including Williams 82, RV8143, ZH10, Y8307, Mc08‐105, and HeFeng55 were used in this study. *N. benthamiana* 17 Wt strain was used for transient expression and protein localization assays. For SMV resistance evaluation, 10 independent Ox‐*SRC4* lines were examined in *N. benthamiana* and 10 independent Ox‐*SRC4* lines were examined in soybean. Based on stable *SRC4* expression and representative SMV resistance phenotypes, *N. benthamiana* Ox‐*SRC4* lines #2 and #22 and soybean Ox‐*SRC4* lines #21 and #22 were selected for detailed analyses. Field experiments were conducted at Nanbin Farm, Sanya, Hainan Province, China (19°32′ N, 109°12′ E; elevation 18 m) under tropical monsoon climate conditions. Stable Ox‐*SRC4* tobacco and soybean plants were generated using the pNC‐Cam33HC‐*SRC4* construct, in which *SRC4* expression was driven by the CaMV 35S promoter and SRC4 was fused to a C‐terminal 2 × HA tag.

### Tobacco transient expression system

*Agrobacterium tumefaciens* GV3101 strain carrying recombinant binary vectors was used to infiltrate tobacco leaves. Cell cultures were resuspended in infiltration buffer (10 mM MES, 10 mM MgCl_2_, 200 μM acetosyringone) and adjusted to an appropriate OD_600_ for infiltration. *Agrobacterium tumefaciens* carrying virus infectious clones (SMV‐GFP) was inoculated in the same way according to the literature (Yan et al., 2022). Each experiment included three biological replicates, and each set of biological replicates contained at least 20–30 technical replicates. After 4–5 dpi, GFP fluorescence was detected by a handheld long‐wave (365 nm) UV lamp. Quantitative analysis of GFP fluorescence intensity was performed using the Fiji software (Schindelin et al., 2012).

### Ca^2+^ and metal cation treatments

Three distinct Ca^2+^ supplementation methods were employed: foliar application, soil incorporation, and root irrigation. For foliar treatments, analytical grade CaCl_2_ (Sigma‐Aldrich, St. Louis, MO, USA; Cat: C1016) was dissolved in sterile deionized water to prepare solutions of specified concentrations (typically 10 mM unless otherwise indicated). Foliar applications were performed twice daily at 08:00 and 18:00 using handheld atomizing sprayers (capacity 500 mL) to ensure uniform coverage. For soil incorporation treatments, 1.5 g CaCl_2_ was thoroughly mixed with 3 L potting soil (Pindstrup Mosebrug A/S, Denmark). For irrigation treatments, 1.5 g CaCl_2_ was dissolved in 1 L sterile deionized water to create 10 mM working solutions applied through root zone watering. Control treatments received equivalent volumes of sterile deionized water. Other metal cations (MgCl_2_, CuSO_4_, ZnCl_2_, FeSO_4_, MnCl_2_, KCl; all analytical grade, Sigma‐Aldrich) were prepared as 10 mM aqueous solutions and applied via foliar spraying using identical protocols. Ca^2+^ channel inhibitors ruthenium red (RR; Sigma‐Aldrich) and gadolinium chloride (GdCl_3_; Sigma‐Aldrich), and chelators EGTA (Sigma‐Aldrich) and EDTA (Sigma‐Aldrich), were prepared as 10 mM stock solutions and applied via foliar spraying following the same schedule as Ca^2+^ treatments.

### Ca^2+^ flux measurement

Real‐time Ca^2+^ translocation was measured using Non‐invasive Micro‐test Technology (NMT Physiolyzer®, Younger USA LLC, Amherst, MA, USA; Model: NMT‐YG‐100). Uniformly developed soybean plants were selected and individual roots were secured at the bottom of petri dishes. Test solution (1.0 mM CaCl_2_, 0.2 mM MES) was added to submerge roots, which were equilibrated for 30 min at 25°C before measurement. Ca^2+^ flux was measured at the root elongation zone (350 μm from root apex) using ion‐selective microelectrodes (tip diameter 2–4 μm) positioned 5 μm from the root surface. Each measurement was recorded for 5 min with 12‐sec intervals using six biological replicates per treatment group. Ca^2+^ flux rates (pico mol·cm^−2^·s^−1^) were acquired and analyzed using imFluxes V3.0 software (Younger USA LLC, Amherst, MA, USA). Negative values indicate influx from external medium into root tissue.

### Fluorescence resonance energy transfer (FRET) analysis

A CFP‐SRC4‐YFP fusion construct was generated by cloning SRC4 into pNC‐Cam2304‐35S vector with cyan fluorescent protein (CFP) fused to the N terminus and yellow fluorescent protein (YFP) to the C terminus. The construct was transiently expressed in tobacco leaves at OD_600_ = 0.5. After 48 h of protein expression, detached leaves were treated with either mock (1 × phosphate‐buffered saline) or Ca^2+^ (10 mM CaCl_2_) solution for 2 h at room temperature. FRET signals were then examined using a confocal laser scanning microscope (Nikon AXR NSPARC). For FRET analysis, CFP was excited at 433 nm (detection at 476 nm) and YFP at 433 nm (detection at 527 nm). The YFP/CFP fluorescence emission ratio was calculated from at least 20 cells per treatment to assess Ca^2+^‐induced conformational changes in SRC4 protein.

### Protein expression and pull‐down assays

For protein expression analysis, total protein was isolated from plant leaves using G10 Lysis buffer (Promega, Madison, WI, USA; E266A) containing protease inhibitor cocktail. Protein concentration was measured with BCA protein assay kit (Solarbio, PC0020). The samples were boiled at 95°C for 10 min, resolved on 10% (W/V) polyacrylamide gel electrophoresis, and immunoblotted to polyvinylidene fluoride membranes. The membranes were blocked, probed with primary antibodies (1:1000 dilution) at 4°C overnight, washed with TBST, incubated with anti‐mouse (Goat) HRP‐linked secondary antibody at room temperature for 2 h, and washed with TBST for 10 min for 3 times. Film exposure was performed after covering with chemiluminescent reagents. The relative molecular weights of protein were determined by loading ColorMixed Protein Marker (11–180 kDa, Solarbio; Lot#PR1910). The SRC4 coding sequence was cloned into pNC‐ET28 vector to generate N‐terminal 6 × His‐tagged fusion protein (His‐SRC4). SMV coat protein (SMV‐CP) was inserted into pGEX4T vector (GE Healthcare, Uppsala, Sweden; Cat: 28‐9545‐49) to produce N‐terminal GST‐tagged fusion (GST‐CP). His‐SRC4 was expressed in *Escherichia coli* BL21 (DE3) and purified using Ni‐NTA agarose resin (Qiagen, Hilden, Germany; Cat: 30210). GST‐CP was expressed and purified using glutathione agarose beads (GSTrap FF, Cytiva; Cat: 17‐5256‐01). For GST pull‐down assays, equal amounts of GST‐CP and GST were immobilized on glutathione affinity beads and incubated with 50 μg of purified His‐SRC4 for 4 h at 4°C with gentle rotation. After incubation, the beads were washed five times with binding buffer, and the retained proteins were analyzed by SDS‐PAGE.

### Seed analysis and phenotyping

Seeds were stored for 1 month post harvest at 4°C in sealed containers before trait determination. Seed morphometric analyses were performed using at least 40 representative dry seeds per genotype. The 100‐seed weight was determined from nine biological replicates per treatment using an analytical balance.

### Fatty acid analysis

Fatty acid compositions were determined by gas chromatography following methyl ester derivatization. Seeds (5 g per accession) were ground using a sample preparation mill (Retsch ZM100, Haan, Germany). Ground samples (300 mg) were transferred to 2‐mL centrifuge tubes containing 1.0 mL n‐hexane (HPLC grade, Sigma‐Aldrich, Cat: 34859) and incubated at 65°C for 20 min with intermittent vortexing. Sodium methoxide solution (1.0 mL, 0.5 M in methanol; Sigma‐Aldrich, Cat: 403067) was added and samples were agitated at 65°C for 10 min to achieve complete methyl esterification. Following centrifugation at 12000 × ***g*** for 2 min, supernatants were analyzed using a GC‐2010 gas chromatograph (Shimadzu Inc., Kyoto, Japan) equipped with a flame ionization detector. Chromatographic separation was performed on an RTX‐WAX column using the following temperature gradient: initial temperature 180°C for 1.5 min, ramped to 210°C at 10°C min^−1^, held at 210°C for 2 min, increased to 220°C at 5°C min^−1^, and maintained at 220°C for 5 min. Nitrogen (99.999% purity) served as carrier gas at 54 mL min^−1^ flow rate with splitless injection mode. Fatty acid concentrations were quantified using area normalization with authentic standards for calibration.

### Statistical analysis

Statistical analyses were performed using GraphPad Prism 7.0 (GraphPad Software, USA) and SPSS 27.0 (IBM, USA). For comparisons between multiple groups, one‐way ANOVA was conducted in GraphPad Prism 7.0, with significance levels indicated by asterisks (*means *P <* 0.05, ***means *P <* 0.001). HSD test was applied via SPSS 27.0 to determine pairwise differences among groups; significant differences (*P <* 0.05) are labeled with distinct lowercase letters in figures.

## Author Contributions

HW and JL conceived and supervised the study. ZK carried out the majority of the experiments. JZ contributed to the seed quality analysis. DM participated in most of the experimental work, and YX managed and analyzed the experimental data. TY, YZ, FY, ZB, FL, and LL were involved in some experiments and data analysis. All authors have read and approved the final manuscript.

## Conflict of Interest

The authors declare no Conflict of Interest.

## Declaration on the Use of generative AI

The authors confirm that no generative artificial intelligence (AI) or AI‐assisted technologies were used in the preparation of this manuscript, including conceptualization, data analysis, figure preparation, or writing of the text. AlphaFold3 was used only as a computational tool for protein structure and interaction prediction during the research process. All scientific interpretation, analysis, and writing were carried out by the human authors, who take full responsibility for the integrity and accuracy of the work.

## Supporting information


**Figure S1.** Representative phenotypes showing Ca^2+^‐enhanced antiviral activity of SRC4 using different supplementation methods.
**Figure S2.** Ca^2+^ concentration optimization for SRC4 antiviral activity.
**Figure S3.** Effects of different metal cations on SRC4 antiviral activity.
**Figure S4.** Identification of SRC4 transgenic tobacco and soybean plants.
**Figure S5.** Representative phenotypes and RT‐qPCR quantification of SRC4 domain truncations in antiviral activity assays.
**Figure S6.** Recombination of SRC7‐TN with SRC4 domains and antiviral activity assays.
**Figure S7.** Recombination of SRC7‐TIR with SRC4 domains and antiviral activity assays.
**Figure S8.** Sequence alignment analysis of SRC4 SNOR domain.
**Figure S9.** Representative phenotypes showing effects of EFh domain mutations on Ca^2+^‐responsiveness.
**Figure S10.** Subcellular localization analysis of SRC4 and truncation variants.
**Figure S11.** BiFC assay for interactions between SRC4 truncations and SMV‐CP.
**Figure S12.** Growth phenotypes of Ox‐SRC4 soybean under different conditions.
**Figure S13.** Proposed mechanism of SRC4‐mediated antiviral activity in soybean.

**Table S1.** Primers.

## Data Availability

All data are available in the main text or the supplementary materials. The nucleotide sequence of gene is available from the Phytozome database (https://phytozome‐next.jgi.doe.gov/) and SoyBase (https://www.soybase.org/). Protein structure predictions were performed using AlphaFold 3 (https://alphafoldserver.com/). All plasmid constructs and biological materials are available from the corresponding authors upon reasonable request. Source data for all figures are provided as supplementary datasets.
