# Supplementary material for: An atypical EF‐hand domain containing resistance protein SRC4 boosts plant immunity in a calcium‐enhanced manner
Source: Plant J. 2026 Sep 15;127(5):e71125. doi: 10.1111/tpj.71125 (PMC13575729; doi:10.1111/tpj.71125)
Supplement: Supplementary file 1 [file TPJ-127-0-s001.docx]

**Figure S1. Representative phenotypes showing Ca^2+^-enhanced antiviral activity of SRC4 using different supplementation methods**

Tobacco (*Nicotiana benthamiana*) leaves were infiltrated with *Agrobacterium tumefaciens* GV3101 (OD_600_=1.0) carrying pNC-Cam2304-35S-SRC4 and co-infected with SMV-GFP. Three different Ca^2+^ supplementation methods were applied: (1) Foliar spraying with 10 mM Ca^2+^ solution twice daily at 8:00 a.m. and 6:00 p.m.; (2) Soil incorporation by adding 1.5 g CaCl_2_ or Ca(NO_3_)_2_ to 3 L culture soil; (3) Root watering with 10 mM Ca^2+^ solution prepared by dissolving 1.5 g CaCl_2_ or Ca(NO_3_)_2_ in 1 L ddH_2_O. Mock treatments received equivalent volumes of ddH_2_O as negative controls. GFP fluorescence was visualized under a hand-held UV lamp (wavelength: 365 nm) at 4 dpi (day post infiltration). Images are representative of at least 15 biological replicates. EV: empty vector control.

**Figure S2. Ca^2+^ concentration optimization for SRC4 antiviral activity**

(A) Representative phenotypes showing the impact of foliar-sprayed Ca^2+^ with different concentrations on SRC4 antiviral activity. CaCl_2_ solutions of varying concentrations: 0.01% (0.9 mM), 0.05% (4.5 mM), 0.1% (9.08 mM), 0.3% (27.2 mM), 0.6% (54.48 mM), and 1.0% (90.8 mM). Tobacco leaves were infiltrated with *Agrobacterium tumefaciens* GV3101 (OD_600_=1.0) carrying *SRC4* expression construct and co-infected with SMV-GFP. GFP fluorescence was captured using a hand-held UV lamp (wavelength: 365 nm) at 4 dpi. Images are representative of at least 15 biological replicates. (B) Quantitative analysis of Ca^2+^ concentration effects on SRC4 antiviral activity. GFP fluorescence intensity was analyzed with Fiji software. The chart shows the fluorescence intensity ratio of SRC4 to EV (empty vector). Data are presented as Mean±SEM. Statistical differences were analyzed by one-way ANOVA, *** means *p*<0.001, n≥15.

**Figure S3. Effects of different metal cations on SRC4 antiviral activity**

(A) Representative phenotypes showing the impact of different metal cations on SRC4 antiviral activity. 10 mM solutions of MgCl_2_, CuSO_4_, ZnCl_2_, FeSO_4_, MnCl_2_, and KCl were applied via leaf spraying. Tobacco leaves were infiltrated with *Agrobacterium tumefaciens* GV3101 (OD_600_=1.0) carrying *SRC4* expression construct and co-infected with SMV-GFP. GFP fluorescence was captured using a hand-held UV lamp (wavelength: 365 nm) at 4 dpi. Images are representative of at least 15 biological replicates. (B) Quantitative analysis of metal cation effects on SRC4 antiviral activity. GFP fluorescence intensity was analyzed with Fiji software. The chart shows the fluorescence intensity ratio of experimental groups to EV. Data are presented as Mean±SEM. Statistical differences were analyzed by one-way ANOVA, *** means *p*<0.001, n≥15.

**Figure S4. Identification of SRC4-transgenic tobacco and soybean plants and RT-qPCR quantification of SMV CP RNA accumulation after SMV infection**

(A–C) Detection and confirmation of Ox-*SRC4* *Nicotiana benthamiana* transgenic lines. (A) RT-qPCR analysis of relative *SRC4* transcript levels in different transgenic lines. (B) Genomic PCR confirmation using *SRC4*-HA-specific primers. Lines #1–#28 represent independent transgenic lines. The expected PCR amplicon size is 972 bp. (C) Immunoblot analysis of HA-tagged SRC4 protein accumulation in selected transgenic lines (#2 and #22) using anti-HA antibodies. Anti-actin antibodies were used as loading controls. (D–F) Detection and confirmation of Ox-*SRC4* soybean (*Glycine max)* transgenic lines. (D) RT-qPCR analysis of relative SRC4 transcript levels in different transgenic lines. (E) Genomic PCR confirmation using *SRC4*-HA-specific primers. Lines #1–#22 represent independent transgenic lines. The expected PCR amplicon size is 972 bp. (F) Immunoblot analysis of HA-tagged SRC4 protein accumulation in selected transgenic lines (#21 and #22) using anti-HA antibodies. Anti-actin antibodies were used as loading controls. (G) RT-qPCR quantification of relative SMV CP RNA accumulation in 17Wt and two independent Ox-*SRC4* *N. benthamiana* lines (#2 and #22) after SMV-GFP infection. Plants were treated by foliar spraying with either 10 mM CaCl_2_ or ddH_2_O (Mock). Total RNA was extracted from infected leaves at 4 dpi. The 17Wt Mock group was set to 1. (H) RT-qPCR quantification of relative SMV CP RNA accumulation in Williams and two independent Ox-*SRC4* soybean lines (#21 and #22) after SMV-N1 inoculation. Plants were treated by foliar spraying with either 10 mM CaCl_2_ or ddH_2_O (Mock). Total RNA was extracted from systemic leaves at 7 dpi. The Williams Mock group was set to 1. For (A), (D), (G), and (H), data are presented as mean ± SD. Three biological replicates were analyzed for each treatment, with three technical replicates per biological replicate. Species-specific Actin genes were used as internal controls for RT-qPCR normalization. Statistical differences were analyzed by one-way ANOVA. *** indicates *p*< 0.001. M, 2K PLUS DNA marker; P, pNC-33HA-SRC4 plasmid positive control; 17Wt, wild-type *N. benthamiana*; Williams, wild-type soybean *cv. Williams 82*; Mock, ddH_2_O treatment; Ca^2+^, 10 mM CaCl_2_ treatment.

**Figure S5. Representative phenotypes of leaves in SRC4 domain truncations and antiviral activity assays**

(A) Tobacco leaves were co-infiltrated with SMV-GFP and different SRC4 domain truncation constructs via *Agrobacterium tumefaciens* GV3101. Mock (ddH_2_O) and Ca^2+^ (10 mM CaCl_2_) treatments were applied via foliar spraying. GFP fluorescence was visualized at 4 dpi. Single domain constructs (N, T, S, E), two-domain constructs (NT, TS, SE), and constructs missing one domain (NTS, TSE) were tested. SRC7-TN served as positive control and EV as negative control. N: NTD domain (1–41 aa); T: TIR domain (42–190 aa); S: SNOR domain (191–242 aa); E: EFh domain (243–305 aa). Images are representative of at least 15 biological replicates for each construct. (B) RT-qPCR analysis of SMV CP RNA accumulation in leaves expressing different SRC4 domain truncation constructs under Mock or Ca^2+^ treatment. Relative SMV CP RNA accumulation was normalized to the EV Mock group. Lower SMV CP RNA accumulation indicates stronger antiviral activity. Data are presented as means ± SD. Statistical significance was determined by one-way ANOVA followed by Tukey’s HSD test in SPSS. Different lowercase letters indicate significant differences at p < 0.05, n = 9. Schematic representations of the corresponding SRC4 domain/truncation constructs are shown in Figure 4A.

**Figure S6. Recombination of SRC7-TN with SRC4 domains and antiviral activity assays**

(A) Representative phenotypes of recombinant constructs. The antiviral SRC7-TN domain is linked to the SRC4 SNOR domain and the EF-hand domain. The recombinant sequence was cloned into the overexpression vector pNC-Cam2304-35S. SRC7-TN: SRC7-TIR+SRC7-NBS. Tobacco leaves were infiltrated with *Agrobacterium tumefaciens* GV3101 (OD_600_=1.0) carrying different recombinant vectors and co-infected with SMV-GFP. Ca^2+^ (10 mM CaCl_2_) was applied exogenously via leaf spray. GFP fluorescence was captured using a hand-held UV lamp (wavelength: 365 nm) at 4 dpi. Images are representative of at least 15 biological replicates. (B) Quantitative analysis of recombinant construct antiviral activity. GFP fluorescence intensity was analyzed with Fiji software. The resistance to SMV-GFP was quantified by the fluorescence intensity ratio of experimental to EV group. Ca^2+^: CaCl_2_ treatment, Mock: ddH_2_O treatment. Data are presented as Mean±SEM. Statistical significance was determined using Tukey's HSD test in SPSS 27.0. Different letters indicate significant differences at *p*<0.05, n≥15.

**Figure S7. Recombination of SRC7-TIR with SRC4 domains and antiviral activity assays**

(A) Representative phenotypes of SRC7-TIR recombinant constructs. The partially antiviral SRC7-TIR domain is linked to the SRC4 SNOR domain and the EF-hand domain. Tobacco leaves were infiltrated with *Agrobacterium tumefaciens* GV3101 (OD_600_=1.0) carrying different recombinant vectors and co-infected with SMV-GFP. Ca^2+^ (10 mM CaCl_2_) was applied exogenously via leaf spray. GFP fluorescence was captured using a hand-held UV lamp (wavelength: 365 nm) at 4 dpi. Images are representative of at least 15 biological replicates. (B) Quantitative analysis of SRC7-TIR recombinant construct antiviral activity. GFP fluorescence intensity was analyzed with Fiji software. Data are presented as Mean±SEM. Statistical significance was determined using Tukey's HSD test in SPSS 27.0. Different letters indicate significant differences at *p*<0.05, n≥15.

**Figure S8. Sequence alignment analysis of SRC4 SNOR domain**

Sequence alignment of SRC4 SNOR and SRC7 NBS domains with other homologous proteins. The SRC4 SNOR domain corresponds to amino acids 191–242, with the amino acid sequence GFHFKYRDEYEYKFIERIVASVSEKINPARIHVADLPVEQESKVQDTHQEQE. Species names are shown on the left. SNOR domain sequence was analyzed by BLAST in the NCBI database (<https://blast.ncbi.nlm.nih.gov/Blast.cgi>). NBS sequences from 30 species were selected for comparison. COBALT (Constraint-based Multiple Alignment Tool) was used to perform the sequence alignment. Black vertical lines indicate conserved amino acid positions across the aligned sequences. The horizontal line above the alignment denotes the NBS region, and the p-loop motif is labeled above the SRC4-SNOR sequence.

**Figure S9. Representative phenotypes showing effects of EFh domain mutations on Ca^2+^-responsiveness**

Tobacco leaves were co-infiltrated with SMV-GFP and EFh domain mutant constructs via *Agrobacterium tumefaciens* GV3101. Mock (ddH_2_O) and Ca^2+^ (10 mM CaCl_2_) treatments were applied via foliar spraying. GFP fluorescence was visualized at 4 dpi. Constructs include SRC7-TIR-EFh Mut1, SRC7-TIR-EFh Mut2, NTS-EFh Mut1, and NTS-EFh Mut2. EFh Mut1: alanine substitutions at -X and -Z positions in the 12-amino acid Ca^2+^-binding loop. EFh Mut2: replacement of SRC4-EFh E2 12-amino acid region with corresponding region from EFh6 domain. SRC7-TN served as positive control and EV as negative control. Images are representative of at least 15 biological replicates for each construct.

**Figure S10. Subcellular localization analysis of SRC4 and truncation variants**

SRC4-eGFP and truncation-eGFP constructs were infiltrated via *Agrobacterium tumefaciens* containing recombinant pNC-Green-SubC vector in tobacco leaves. After 2 days, tobacco leaf fluorescence was captured under the following settings: objective lens magnification of 20×; Pinhole remains at 1 AU; Master Gain 500–700. mCherry: Histone3-mCherry for labeling cell nucleus with red fluorescence, LTI6b-mCherry for labeling cell membrane with red fluorescence. Scale bar: 50 μm.

**Figure S11. BiFC assay for interactions between SRC4 truncations and SMV-CP**

BiFC assays were conducted to examine interactions between SRC4 truncation variants and SMV coat protein. nVenus + cVenus served as a negative control. After 2 days, Venus signal (excitation at 514 nm and fluorescence detection at 519–620 nm) was captured under the following settings: objective lens magnification of 20×; Pinhole remains at 1 AU; Master Gain 500–700. mCherry: Histone3-mCherry for labeling cell nucleus with red fluorescence, LTI6b-mCherry for labeling cell membrane with red fluorescence. Scale bar: 50 μm. Because the representative optical sections were selected to clearly visualize the plasma-membrane-associated Venus signals, Histone3-mCherry-labelled nuclei outside the selected focal plane may appear diffuse in some panels.

**Figure S12. Growth phenotypes of Ox-*SRC4* soybean under different conditions**

(A) Frontal and top views of soybean plants at different developmental stages. Images were captured at 30 and 70 days after planting. The plants were grown in a standard greenhouse with a 10-hour daily photoperiod extension using broad-spectrum LED panels. The ambient temperature was precisely maintained at 28.0°C. Images display the overall plant morphology of Williams (left) and Ox-*SRC4* (right). Scale bar: 5 cm. (B) Growth phenotypes at approximately 45 and 90 days. Plants were cultivated in a latosol field (19°32′N, 109°12′E; elevation 18 m) at Nanbin Farm, Sanya, Hainan Province, China. The field trial was conducted under tropical monsoon climate conditions.

**Figure S13. Proposed model of SRC4-mediated antiviral activity in soybean.** The TIR domain confers basal antiviral activity, and the adjacent SNOR region enhances TIR-dependent antiviral activity and contributes to SRC4 self-association. The EFh domain interacts with SMV CP and regulates TIR–SNOR-mediated antiviral activity in a Ca^2+^-responsive manner. In the proposed model, Ca^2+^ binding to the EFh domain induces a conformational change in SRC4, thereby further enhancing its antiviral activity. Thus, the coordinated functions of the TIR, SNOR, and EFh domains enable SRC4 to integrate SMV recognition with Ca^2+^-responsive immune regulation. N: NTD domain; T: TIR domain; S: SNOR domain; E: EFh domain.
