# Supplementary material for: An atypical EF‐hand domain containing resistance protein SRC4 boosts plant immunity in a calcium‐enhanced manner
Source: Plant J. 2026 Sep 15;127(5):e71125. doi: 10.1111/tpj.71125 (PMC13575729; doi:10.1111/tpj.71125)
Supplement: Supplementary file 2 [file TPJ-127-0-s002.docx]

**Supplemental Table S1.** Primers used in this study.

| **Vector** | **Gene** | **Orientation** | **5′-3′ primer sequence** | **Purpose** |
| --- | --- | --- | --- | --- |
| pNC-Cam2304-35S | SRC4 | P001 (Fwd) | agtggtctctgtccagtcctATGAATGAGAAAAAGCAAGATA | Plant expression |
|  |  | P002 (Rev) | ggtctcagcagaccacaagtAATTTTCATAGTCTTAACAAAT |  |
| pNC-Cam2304-35S | SRC7-TN | P003 (Fwd) | agtggtctctgtccagtcctATGGCTGCAACAACACGTTCC | Plant expression |
|  |  | P004 (Rev) | ggtctcagcagaccacaagtTTATCTTTCCCGTATTGTAACATCAAA |  |
| pNC-Cam33HC | SRC4-2xHA | P001 (Fwd) | agtggtctctgtccagtcctATGAATGAGAAAAAGCAAGATA | Plant expression |
|  |  | P002 (Rev) | ggtctcagcagaccacaagtAATTTTCATAGTCTTAACAAAT |  |
| pNC-Cam2304-35S | CFP-SRC4-YFP | P007 (Fwd) | agtggtctctgtccagtcctATGGTGAGCAAGGGCGAGGAGC | FRET assay |
|  |  | P008 (Rev) | ggtctcagcagaccacaagtcttgtacagctcgtccatgccgaga |  |
| pNC-Cam2304-35S | N | P001 (Fwd) | agtggtctctgtccagtcctATGAATGAGAAAAAGCAAGATA | Domain truncation / plant expression |
|  |  | P013 (Rev) | ggtctcagcagaccacaagtTATTAGATGAAAAAAGTTTAGAATGAAACT |  |
| pNC-Cam2304-35S | T | P014 (Fwd) | agtggtctctgtccagtcctATGGCTGCAACAACACGTTCACGTGC | Domain truncation / plant expression |
|  |  | P015 (Rev) | ggtctcagcagaccacaagtAGACAAGTCAGCAACTTGACG |  |
| pNC-Cam2304-35S | S | P016 (Fwd) | agtggtctctgtccagtcctGGCTTTCATTTCAAATACAGA | Domain truncation / plant expression |
|  |  | P017 (Rev) | ggtctcagcagaccacaagtGATACCCATCAAGAGCAAGAA |  |
| pNC-Cam2304-35S | E | P018 (Fwd) | agtggtctctgtccagtcctTACAGAGAGGCTTTCAAGGTGT | Domain truncation / plant expression |
|  |  | P019 (Rev) | ggtctcagcagaccacaagtAATTTTCATAGTCTTAACAAA |  |
| pNC-Cam2304-35S | NT | P001 (Fwd) | agtggtctctgtccagtcctATGAATGAGAAAAAGCAAGATA | Domain truncation / plant expression |
|  |  | P015 (Rev) | ggtctcagcagaccacaagtAGACAAGTCAGCAACTTGACG |  |
| pNC-Cam2304-35S | TS | P014 (Fwd) | agtggtctctgtccagtcctATGGCTGCAACAACACGTTCACGTGC | Domain truncation / plant expression |
|  |  | P017 (Rev) | ggtctcagcagaccacaagtGATACCCATCAAGAGCAAGAA |  |
| pNC-Cam2304-35S | SE | P020 (Fwd) | agtggtctctgtccagtcctGGCTTTCATTTCAAATACA | Domain truncation / plant expression |
|  |  | P002 (Rev) | ggtctcagcagaccacaagtAATTTTCATAGTCTTAACAAAT |  |
| pNC-Cam2304-35S | NTS | P001 (Fwd) | agtggtctctgtccagtcctATGAATGAGAAAAAGCAAGATA | Domain truncation / plant expression |
|  |  | P017 (Rev) | ggtctcagcagaccacaagtGATACCCATCAAGAGCAAGAA |  |
| pNC-Cam2304-35S | TSE | P014 (Fwd) | agtggtctctgtccagtcctATGGCTGCAACAACACGTTCACGTGC | Domain truncation / plant expression |
|  |  | P002 (Rev) | ggtctcagcagaccacaagtAATTTTCATAGTCTTAACAAAT |  |
| pNC-Cam2304-35S | NTE1E2 | P001 (Fwd) | agtggtctctgtccagtcctATGAATGAGAAAAAGCAAGATA | Domain truncation / plant expression |
|  |  | P019 (Rev) | ggtctcagcagaccacaagtAATTTTCATAGTCTTAACAAA |  |
| pNC-Cam2304-35S | NTE1 | P001 (Fwd) | agtggtctctgtccagtcctATGAATGAGAAAAAGCAAGATA | Domain truncation / plant expression |
|  |  | P023 (Rev) | ggtctcagcagaccacaagtACCCAGTTTGATCAGAACTT |  |
| pNC-Cam2304-35S | NTE2 | P001 (Fwd) | agtggtctctgtccagtcctATGAATGAGAAAAAGCAAGATA | Domain truncation / plant expression |
|  |  | P024 (Rev) | ggtctcagcagaccacaagtTTAAATTTTCATAGTCTTAAC |  |
| pNC-Cam2304-35S | NTSE1 | P001 (Fwd) | agtggtctctgtccagtcctATGAATGAGAAAAAGCAAGATA | Domain truncation / plant expression |
|  |  | P023 (Rev) | ggtctcagcagaccacaagtACCCAGTTTGATCAGAACTT |  |
| pNC-Cam2304-35S | NTSE2 | P001 (Fwd) | agtggtctctgtccagtcctATGAATGAGAAAAAGCAAGATA | Domain truncation / plant expression |
|  |  | P024 (Rev) | ggtctcagcagaccacaagtTTAAATTTTCATAGTCTTAAC |  |
| pNC-Cam2304-35S | SRC4 / NTSE1E2 | P001 (Fwd) | agtggtctctgtccagtcctATGAATGAGAAAAAGCAAGATA | Domain truncation / plant expression |
|  |  | P002 (Rev) | ggtctcagcagaccacaagtAATTTTCATAGTCTTAACAAAT |  |
| pNC-Cam2304-35S | TSE1E2 | P014 (Fwd) | agtggtctctgtccagtcctATGGCTGCAACAACACGTTCACGTGC | Domain truncation / plant expression |
|  |  | P002 (Rev) | ggtctcagcagaccacaagtAATTTTCATAGTCTTAACAAAT |  |
| pNC-Cam2304-35S | TE1 | P014 (Fwd) | agtggtctctgtccagtcctATGGCTGCAACAACACGTTCACGTGC | Domain truncation / plant expression |
|  |  | P023 (Rev) | ggtctcagcagaccacaagtACCCAGTTTGATCAGAACTT |  |
| pNC-Cam2304-35S | TE2 | P014 (Fwd) | agtggtctctgtccagtcctATGGCTGCAACAACACGTTCACGTGC | Domain truncation / plant expression |
|  |  | P024 (Rev) | ggtctcagcagaccacaagtTTAAATTTTCATAGTCTTAAC |  |
| pNC-Cam2304-35S | TSE1 | P014 (Fwd) | agtggtctctgtccagtcctATGGCTGCAACAACACGTTCACGTGC | Domain truncation / plant expression |
|  |  | P023 (Rev) | ggtctcagcagaccacaagtACCCAGTTTGATCAGAACTT |  |
| pNC-Cam2304-35S | TSE2 | P014 (Fwd) | agtggtctctgtccagtcctATGGCTGCAACAACACGTTCACGTGC | Domain truncation / plant expression |
|  |  | P024 (Rev) | ggtctcagcagaccacaagtTTAAATTTTCATAGTCTTAAC |  |
| pNC-Cam2304-35S | TE1E2 | P014 (Fwd) | agtggtctctgtccagtcctATGGCTGCAACAACACGTTCACGTGC | Domain truncation / plant expression |
|  |  | P019 (Rev) | ggtctcagcagaccacaagtAATTTTCATAGTCTTAACAAA |  |
| pNC-Cam2304-35S | TS52 | P014 (Fwd) | agtggtctctgtccagtcctATGGCTGCAACAACACGTTCACGTGC | Site-directed mutagenesis / plant expression |
|  |  | P017 (Rev) | ggtctcagcagaccacaagtGATACCCATCAAGAGCAAGAA |  |
| pNC-Cam2304-35S | TS40 | P014 (Fwd) | agtggtctctgtccagtcctATGGCTGCAACAACACGTTCACGTGC | Site-directed mutagenesis / plant expression |
|  |  | P032 (Rev) | ggtctcagcagaccacaagtTTGTTCAACAGGCAGATC |  |
| pNC-Cam2304-35S | TS30 | P014 (Fwd) | agtggtctctgtccagtcctATGGCTGCAACAACACGTTCACGTGC | Site-directed mutagenesis / plant expression |
|  |  | P033 (Rev) | ggtctcagcagaccacaagtTCTAGCAGGATTAATCT |  |
| pNC-Cam2304-35S | TS20 | P014 (Fwd) | agtggtctctgtccagtcctATGGCTGCAACAACACGTTCACGTGC | Site-directed mutagenesis / plant expression |
|  |  | P034 (Rev) | ggtctcagcagaccacaagtCGCAACAATTCTCTCAATAAAC |  |
| pNC-Cam2304-35S | TS10 | P014 (Fwd) | agtggtctctgtccagtcctATGGCTGCAACAACACGTTCACGTGC | Site-directed mutagenesis / plant expression |
|  |  | P035 (Rev) | ggtctcagcagaccacaagtATATTCATCTCTGTATTTG |  |
| pNC-Cam2304-35S | TSm | P014 (Fwd) | agtggtctctgtccagtcctATGGCTGCAACAACACGTTCACGTGC | Site-directed mutagenesis / plant expression |
|  |  | P017 (Rev) | ggtctcagcagaccacaagtGATACCCATCAAGAGCAAGAA |  |
| pNC-Cam2304-35S | SRC4m | P001 (Fwd) | agtggtctctgtccagtcctATGAATGAGAAAAAGCAAGATA | Site-directed mutagenesis / plant expression |
|  |  | P002 (Rev) | ggtctcagcagaccacaagtAATTTTCATAGTCTTAACAAAT |  |
| pNC-Cam2304-35S | Sm | P016 (Fwd) | agtggtctctgtccagtcctGGCTTTCATTTCAAATACAGA | Site-directed mutagenesis / plant expression |
|  |  | P017 (Rev) | ggtctcagcagaccacaagtGATACCCATCAAGAGCAAGAA |  |
| pNC-GADT7 | AD-SRC4 | P001 (Fwd) | agtggtctctgtccagtcctATGAATGAGAAAAAGCAAGATA | Yeast two-hybrid assay |
|  |  | P038 (Rev) | ggtctcagcagaccacaagtTTAAATTTTCATAGTCTTAACAAAT |  |
| pNC-GBKT7 | BD-SRC4 | P001 (Fwd) | agtggtctctgtccagtcctATGAATGAGAAAAAGCAAGATA | Yeast two-hybrid assay |
|  |  | P038 (Rev) | ggtctcagcagaccacaagtTTAAATTTTCATAGTCTTAACAAAT |  |
| pNC-GADT7 | AD-S | P016 (Fwd) | agtggtctctgtccagtcctGGCTTTCATTTCAAATACAGA | Yeast two-hybrid assay |
|  |  | P017 (Rev) | ggtctcagcagaccacaagtGATACCCATCAAGAGCAAGAA |  |
| pNC-GBKT7 | BD-S | P016 (Fwd) | agtggtctctgtccagtcctGGCTTTCATTTCAAATACAGA | Yeast two-hybrid assay |
|  |  | P017 (Rev) | ggtctcagcagaccacaagtGATACCCATCAAGAGCAAGAA |  |
| pNC-GADT7 | AD-SRC4T | P014 (Fwd) | agtggtctctgtccagtcctATGGCTGCAACAACACGTTCACGTGC | Yeast two-hybrid assay |
|  |  | P015 (Rev) | ggtctcagcagaccacaagtAGACAAGTCAGCAACTTGACG |  |
| pNC-GBKT7 | BD-SRC4T | P014 (Fwd) | agtggtctctgtccagtcctATGGCTGCAACAACACGTTCACGTGC | Yeast two-hybrid assay |
|  |  | P015 (Rev) | ggtctcagcagaccacaagtAGACAAGTCAGCAACTTGACG |  |
| pNC-GADT7 | AD-Sm | P016 (Fwd) | agtggtctctgtccagtcctGGCTTTCATTTCAAATACAGA | Yeast two-hybrid assay |
|  |  | P017 (Rev) | ggtctcagcagaccacaagtGATACCCATCAAGAGCAAGAA |  |
| pNC-GBKT7 | BD-Sm | P016 (Fwd) | agtggtctctgtccagtcctGGCTTTCATTTCAAATACAGA | Yeast two-hybrid assay |
|  |  | P017 (Rev) | ggtctcagcagaccacaagtGATACCCATCAAGAGCAAGAA |  |
| pNC-GADT7 | AD-SRC4m | P001 (Fwd) | agtggtctctgtccagtcctATGAATGAGAAAAAGCAAGATA | Yeast two-hybrid assay |
|  |  | P038 (Rev) | ggtctcagcagaccacaagtTTAAATTTTCATAGTCTTAACAAAT |  |
| pNC-GBKT7 | BD-SRC4m | P001 (Fwd) | agtggtctctgtccagtcctATGAATGAGAAAAAGCAAGATA | Yeast two-hybrid assay |
|  |  | P038 (Rev) | ggtctcagcagaccacaagtTTAAATTTTCATAGTCTTAACAAAT |  |
| pNC-BiFC-Vnc | SRC4-nVenus | P001 (Fwd) | agtggtctctgtccagtcctATGAATGAGAAAAAGCAAGATA | Bimolecular fluorescence complementation |
|  |  | P002 (Rev) | ggtctcagcagaccacaagtAATTTTCATAGTCTTAACAAAT |  |
| pNC-BiFC-Vcc | SRC4-cVenus | P001 (Fwd) | agtggtctctgtccagtcctATGAATGAGAAAAAGCAAGATA | Bimolecular fluorescence complementation |
|  |  | P002 (Rev) | ggtctcagcagaccacaagtAATTTTCATAGTCTTAACAAAT |  |
| pNC-BiFC-Vnc | SRC4-S-nVenus | P016 (Fwd) | agtggtctctgtccagtcctGGCTTTCATTTCAAATACAGA | Bimolecular fluorescence complementation |
|  |  | P017 (Rev) | ggtctcagcagaccacaagtGATACCCATCAAGAGCAAGAA |  |
| pNC-BiFC-Vcc | SRC4-S-cVenus | P016 (Fwd) | agtggtctctgtccagtcctGGCTTTCATTTCAAATACAGA | Bimolecular fluorescence complementation |
|  |  | P017 (Rev) | ggtctcagcagaccacaagtGATACCCATCAAGAGCAAGAA |  |
| pNC-BiFC-Vnc | SRC4-T-nVenus | P014 (Fwd) | agtggtctctgtccagtcctATGGCTGCAACAACACGTTCACGTGC | Bimolecular fluorescence complementation |
|  |  | P015 (Rev) | ggtctcagcagaccacaagtAGACAAGTCAGCAACTTGACG |  |
| pNC-BiFC-Vcc | SRC4-T-cVenus | P014 (Fwd) | agtggtctctgtccagtcctATGGCTGCAACAACACGTTCACGTGC | Bimolecular fluorescence complementation |
|  |  | P015 (Rev) | ggtctcagcagaccacaagtAGACAAGTCAGCAACTTGACG |  |
| pNC-BiFC-Vnc | SRC4m-nVenus | P001 (Fwd) | agtggtctctgtccagtcctATGAATGAGAAAAAGCAAGATA | Bimolecular fluorescence complementation |
|  |  | P002 (Rev) | ggtctcagcagaccacaagtAATTTTCATAGTCTTAACAAAT |  |
| pNC-BiFC-Vcc | SRC4m-cVenus | P001 (Fwd) | agtggtctctgtccagtcctATGAATGAGAAAAAGCAAGATA | Bimolecular fluorescence complementation |
|  |  | P002 (Rev) | ggtctcagcagaccacaagtAATTTTCATAGTCTTAACAAAT |  |
| pNC-BiFC-Vnc | Sm-nVenus | P016 (Fwd) | agtggtctctgtccagtcctGGCTTTCATTTCAAATACAGA | Bimolecular fluorescence complementation |
|  |  | P017 (Rev) | ggtctcagcagaccacaagtGATACCCATCAAGAGCAAGAA |  |
| pNC-BiFC-Vcc | Sm-cVenus | P016 (Fwd) | agtggtctctgtccagtcctGGCTTTCATTTCAAATACAGA | Bimolecular fluorescence complementation |
|  |  | P017 (Rev) | ggtctcagcagaccacaagtGATACCCATCAAGAGCAAGAA |  |
| pNC-Green-SubC | SRC4(NTSE1E2)-eGFP | P001 (Fwd) | agtggtctctgtccagtcctATGAATGAGAAAAAGCAAGATA | Subcellular localization |
|  |  | P002 (Rev) | ggtctcagcagaccacaagtAATTTTCATAGTCTTAACAAAT |  |
| pNC-Green-SubC | NTSE1-eGFP | P001 (Fwd) | agtggtctctgtccagtcctATGAATGAGAAAAAGCAAGATA | Subcellular localization |
|  |  | P023 (Rev) | ggtctcagcagaccacaagtACCCAGTTTGATCAGAACTT |  |
| pNC-Green-SubC | NTS-eGFP | P001 (Fwd) | agtggtctctgtccagtcctATGAATGAGAAAAAGCAAGATA | Subcellular localization |
|  |  | P017 (Rev) | ggtctcagcagaccacaagtGATACCCATCAAGAGCAAGAA |  |
| pNC-Green-SubC | NT-eGFP | P001 (Fwd) | agtggtctctgtccagtcctATGAATGAGAAAAAGCAAGATA | Subcellular localization |
|  |  | P015 (Rev) | ggtctcagcagaccacaagtAGACAAGTCAGCAACTTGACG |  |
| pNC-Green-SubC | EFh(E1E2)-eGFP | P018 (Fwd) | agtggtctctgtccagtcctTACAGAGAGGCTTTCAAGGTGT | Subcellular localization |
|  |  | P019 (Rev) | ggtctcagcagaccacaagtAATTTTCATAGTCTTAACAAA |  |
| pNC-BiFC-Vnc | SRC4(NTSE1E2)-nVenus | P001 (Fwd) | agtggtctctgtccagtcctATGAATGAGAAAAAGCAAGATA | Bimolecular fluorescence complementation |
|  |  | P002 (Rev) | ggtctcagcagaccacaagtAATTTTCATAGTCTTAACAAAT |  |
| pNC-BiFC-Vcc | CP-cVenus | P039 (Fwd) | agtggtctctgtccagtccttcaggcaaggagaaggaag | Bimolecular fluorescence complementation |
|  |  | P040 (Rev) | ggtctcagcagaccacaagtTCActgcggtgggcccatg |  |
| pNC-BiFC-Vnc | NTSE1-nVenus | P001 (Fwd) | agtggtctctgtccagtcctATGAATGAGAAAAAGCAAGATA | Bimolecular fluorescence complementation |
|  |  | P023 (Rev) | ggtctcagcagaccacaagtACCCAGTTTGATCAGAACTT |  |
| pNC-BiFC-Vnc | NTS-nVenus | P001 (Fwd) | agtggtctctgtccagtcctATGAATGAGAAAAAGCAAGATA | Bimolecular fluorescence complementation |
|  |  | P017 (Rev) | ggtctcagcagaccacaagtGATACCCATCAAGAGCAAGAA |  |
| pNC-BiFC-Vnc | NT-nVenus | P001 (Fwd) | agtggtctctgtccagtcctATGAATGAGAAAAAGCAAGATA | Bimolecular fluorescence complementation |
|  |  | P015 (Rev) | ggtctcagcagaccacaagtAGACAAGTCAGCAACTTGACG |  |
| pNC-BiFC-Vnc | E1E2(EFh)-nVenus | P018 (Fwd) | agtggtctctgtccagtcctTACAGAGAGGCTTTCAAGGTGT | Bimolecular fluorescence complementation |
|  |  | P019 (Rev) | ggtctcagcagaccacaagtAATTTTCATAGTCTTAACAAA |  |
| pNC-Cam2304-35S | SRC7-TIR-EFh7+7 | P003 (Fwd) | agtggtctctgtccagtcctATGGCTGCAACAACACGTTCC | EF-hand construct / plant expression |
|  |  | P041 (Rev) | ggtctcagcagaccacaagtTCATCCAATAATCATCATCAT |  |
| pNC-Cam2304-35S | SRC7-TIR-EFh6 | P003 (Fwd) | agtggtctctgtccagtcctATGGCTGCAACAACACGTTCC | EF-hand construct / plant expression |
|  |  | P047 (Rev) | ggtctcagcagaccacaagtTAAGCTATTAGGAATGGC |  |
| pNC-Cam2304-35S | SRC7-TIR-EFh1 | P003 (Fwd) | agtggtctctgtccagtcctATGGCTGCAACAACACGTTCC | EF-hand construct / plant expression |
|  |  | P053 (Rev) | ggtctcagcagaccacaagtTAATTTTGGCAGA |  |
| pNC-Cam2304-35S | SRC7-TIR-EFh2 | P003 (Fwd) | agtggtctctgtccagtcctATGGCTGCAACAACACGTTCC | EF-hand construct / plant expression |
|  |  | P057 (Rev) | ggtctcagcagaccacaagtTCCACTCAAATCCCTTAAAG |  |
| pNC-Cam2304-35S | SRC7-TIR-EFh5+1 | P003 (Fwd) | agtggtctctgtccagtcctATGGCTGCAACAACACGTTCC | EF-hand construct / plant expression |
|  |  | P063 (Rev) | ggtctcagcagaccacaagtAATCTCAGTCATTAAGGCCTTG |  |
| pNC-Cam2304-35S | SRC7-TIR-EFh8 | P003 (Fwd) | agtggtctctgtccagtcctATGGCTGCAACAACACGTTCC | EF-hand construct / plant expression |
|  |  | P069 (Rev) | ggtctcagcagaccacaagtTACAAGCAGGCCATCTCCATTCAAAT |  |
| pNC-Cam2304-35S | NTS-EFh Mut1 | P001 (Fwd) | agtggtctctgtccagtcctATGAATGAGAAAAAGCAAGATA | Site-directed mutagenesis / plant expression |
|  |  | P002 (Rev) | ggtctcagcagaccacaagtAATTTTCATAGTCTTAACAAAT |  |
| pNC-Cam2304-35S | NTS-EFh Mut2 | P001 (Fwd) | agtggtctctgtccagtcctATGAATGAGAAAAAGCAAGATA | Site-directed mutagenesis / plant expression |
|  |  | P002 (Rev) | ggtctcagcagaccacaagtAATTTTCATAGTCTTAACAAAT |  |
| pNC-Cam2304-35S | SRC7-TIR-EFh Mut1 | P003 (Fwd) | agtggtctctgtccagtcctATGGCTGCAACAACACGTTCC | Site-directed mutagenesis / plant expression |
|  |  | P041 (Rev) | ggtctcagcagaccacaagtTCATCCAATAATCATCATCAT |  |
| pNC-Cam2304-35S | SRC7-TIR-EFh Mut2 | P003 (Fwd) | agtggtctctgtccagtcctATGGCTGCAACAACACGTTCC | Site-directed mutagenesis / plant expression |
|  |  | P041 (Rev) | ggtctcagcagaccacaagtTCATCCAATAATCATCATCAT |  |
| pNC-GADT7 | AD-P1 | P079 (Fwd) | agtggtctctgtccagtcctgcaacaatcatgattgg | Yeast two-hybrid assay |
|  |  | P080 (Rev) | ggtctcagcagaccacaagttagtgctgaatatcctccat |  |
| pNC-GADT7 | AD-P3 | P081 (Fwd) | agtggtctctgtccagtcctggtgaagtgcaacagagaat | Yeast two-hybrid assay |
|  |  | P082 (Rev) | ggtctcagcagaccacaagtctgtgcggagacatcttct |  |
| pNC-GADT7 | AD-6K1 | P083 (Fwd) | agtggtctctgtccagtcctccaaaaaagaagagaaaggtagaagaccccgccaaaacagcaactcaatt | Yeast two-hybrid assay |
|  |  | P084 (Rev) | ggtctcagcagaccacaagtctgcactttaacatcctcac |  |
| pNC-GADT7 | AD-6K2 | P085 (Fwd) | agtggtctctgtccagtcctccaaaaaagaagagaaaggtagaagaccccagcaagcatgaggtgagtaaa | Yeast two-hybrid assay |
|  |  | P086 (Rev) | ggtctcagcagaccacaagtttgagttgatactggttcacg |  |
| pNC-GADT7 | AD-NIa-Pro | P087 (Fwd) | agtggtctctgtccagtcctagcaaatctgtttacaaagga | Yeast two-hybrid assay |
|  |  | P088 (Rev) | ggtctcagcagaccacaagtttgaactgtcactgtgtttc |  |
| pNC-GADT7 | AD-NIa-VPg | P089 (Fwd) | agtggtctctgtccagtcctggaaagaagaggcagatac | Yeast two-hybrid assay |
|  |  | P090 (Rev) | ggtctcagcagaccacaagtttccatttcaaccctttcttt |  |
| pNC-GADT7 | AD-CP | P091 (Fwd) | agtggtctctgtccagtcctccaaaaaagaagagaaaggtagaagacccctcaggcaaggagaaggaag | Yeast two-hybrid assay |
|  |  | P092 (Rev) | ggtctcagcagaccacaagtctgcggtgggcccatg |  |
| pNC-GADT7 | AD-CI | P093 (Fwd) | agtggtctctgtccagtcctccaaaaaagaagagaaaggtagaagaccccagtcttgatgaaattcaaagc | Yeast two-hybrid assay |
|  |  | P094 (Rev) | ggtctcagcagaccacaagtctgtagttggactgcattta |  |
| pNC-GADT7 | AD-HC-Pro | P095 (Fwd) | agtggtctctgtccagtccttcccaaaatcctgaagctca | Yeast two-hybrid assay |
|  |  | P096 (Rev) | ggtctcagcagaccacaagtaccaactctgtagaatttcat |  |
| pNC-GADT7 | AD-NIb | P097 (Fwd) | agtggtctctgtccagtcctgggagaaaggaaagatggg | Yeast two-hybrid assay |
|  |  | P098 (Rev) | ggtctcagcagaccacaagtttgtaaggacactgattcaca |  |
| pGEX-4T-1 | GST-CP | P099 (Fwd) | gatctggttccgcgtggatccccgtcaggcaaggagaaggaag | Protein expression and pull-down assay |
|  |  | P100 (Rev) | tcagtcagtcacgatgcggccgcTCActgcggtgggcccatg |  |
| pNC-ET28 | His-SRC4 | P001 (Fwd) | agtggtctctgtccagtcctATGAATGAGAAAAAGCAAGATA | Protein expression and pull-down assay |
|  |  | P038 (Rev) | ggtctcagcagaccacaagtTTAAATTTTCATAGTCTTAACAAAT |  |

** Lowercase bases denote NC/vector-homology arms where applicable.*
